# GINS2 regulates temozolomide chemosensitivity via the EGR1/ECT2 axis in gliomas

**DOI:** 10.1038/s41419-024-06586-w

**Published:** 2024-03-11

**Authors:** Hua He, Lu Liang, Shiyao Jiang, Yueying Liu, Jingjing Huang, Xiaoyan Sun, Yi Li, Yiqun Jiang, Li Cong

**Affiliations:** 1 The Key Laboratory of Model Animal and Stem Cell Biology in Hunan Province, Hunan Normal University, Changsha, 410013 Hunan China; 2 School of Medicine, Hunan Normal University, Changsha, 410013 Hunan China

**Keywords:** Tumour biomarkers, CNS cancer

## Abstract

Temozolomide (TMZ), a DNA alkylating agent, has become the primary treatment for glioma, the most common malignancy of the central nervous system. Although TMZ-containing regimens produce significant clinical response rates, some patients inevitably suffer from inferior treatment outcomes or disease relapse, likely because of poor chemosensitivity of glioma cells due to a robust DNA damage response (DDR). GINS2, a subunit of DNA helicase, contributes to maintaining genomic stability and is highly expressed in various cancers, promoting their development. Here, we report that GINS2 was upregulated in TMZ-treated glioma cells and co-localized with γH2AX, indicating its participation in TMZ-induced DDR. Furthermore, GINS2 regulated the malignant phenotype and TMZ sensitivity of glioma cells, mostly by promoting DNA damage repair by affecting the mRNA stability of early growth response factor 1 (EGR1), which in turn regulates the transcription of epithelial cell-transforming sequence 2 (ECT2). We constructed a GINS2–EGR1–ECT2 prognostic model, which accurately predicted patient survival. Further, we screened Palbociclib/BIX-02189 which dampens GINS2 expression and synergistically inhibits glioma cell proliferation with TMZ. These findings delineate a novel mechanism by which GINS2 regulates the TMZ sensitivity of glioma cells and propose a promising combination therapy to treat glioma.

## Introduction

Gliomas, the most common primary malignant tumors of the central nervous system, are characterized by rapid growth, high degree of infiltration, and high likelihood of relapse [1, 2]. Based on their pathological histology, gliomas are classified as glioblastoma (GBM), astrocytoma and oligodendroglioma [3]. A combination of surgery, radiotherapy, and chemotherapy is commonly used to manage gliomas. Temozolomide (TMZ), the first-line chemotherapeutic agent for treating glioma, alkylates the guanine O^6^ site of DNA, leading to the introduction of base mismatches during DNA replication and ultimately inducing DNA double-stranded breaks (DSBs) [4, 5]. Although some patients respond effectively to TMZ treatment, the median survival time is still relatively low [6]. Intrinsic resistance or a decrease in sensitivity post-treatment may impede the efficacy of TMZ treatment. Therefore, enhancing the sensitivity to TMZ is vital for maintaining its anticancer efficacy against glioma.

To respond to DNA damage, multiple biological processes, including DNA repair and the cell cycle, are coordinated into an overarching DNA damage response (DDR). Dysregulation of the DDR is not only associated with susceptibility to carcinogenesis but may also contribute to tumor resistance by repairing chemotherapy- or radiotherapy-induced DNA damage [7, 8]. The chemoresistance of gliomas to TMZ is largely attributed to the direct repair of DNA damage by O^6^-methylguanine-DNA methyltransferase, but it is not the only molecular mechanism in action. Pathways that mediate DSB repair, such as base excision repair, mismatch repair, homologous recombination, and non-homologous end-joining, may also influence TMZ sensitivity [9, 10].

As a component of the cell division cycle protein 45–minichromosome maintenance (MCM) complex–GINS (CMG) helicase, the GINS complex binds to DNA and participates in DNA replication. It consists of four conserved proteins: GINS1 (Psf1), GINS2 (Psf2), GINS3 (Psf3), and GINS4 (Sld5) [11–13]. GINS2 was shown to be associated with the progression of several cancers, including lung cancer [14, 15], breast cancer [16], cervical cancer [17], leukemia [18], and thyroid cancer [19], by manipulating several signaling pathways, such as the phosphoinositide 3-kinase/AKT/mammalian target of rapamycin (mTOR) [20], P53/growth arrest and DNA damage-inducible protein 45 alpha [14], and mitogen-activated protein kinase kinase/extracellular signal-regulated kinase pathways [15]. In addition, suppressing the expression of GINS2 significantly reduced the proliferation and tumorigenicity of glioma cells, probably via the action of cell cycle-related genes [21]. Therefore, GINS2 may be an essential biomarker for the diagnosis and prognosis of glioma.

Epithelial cell-transforming sequence 2 (ECT2) is a protein containing the breast cancer gene 1 (BRCA1) C-terminal (BRCT) structural domain and a guanine nucleotide exchange factor for Ras homolog family member A (RhoA) [22–24]. ECT2 positively regulates the activation of RhoA, which participates in cell adhesion, transformation, and division. Moreover, RhoA responds to DNA damage by regulating the p38–mitogen-activated protein kinase pathway and inducing cell cycle arrest via Rho-associated protein kinase for DNA repair. Proteins containing the BRCT structural domain are usually critical regulators of DNA damage signaling [25]. ECT2 participates in DSB repair by promoting the assembly of BRCA1, the core factor of homologous recombination, and KU70, a non-homologous end-joining factor, on damaged chromatin [26]. In the presence of DNA damage, ECT2, via its BRCT structural domain, was shown to interact with mitogen-activated protein kinase-associated protein 1 (Sin1), a core component of TOR complex 2, to activate the downstream mTOR complex 2/AKT signaling pathway, stimulating the repair of DNA damage in various cancer and non-cancer cells [27]. These studies suggest that high ECT2 expression may enhance tumor resistance to radiotherapy or chemotherapy via the DDR.

The Connectivity Map (CMap) is a database that correlates perturbagens, gene expression, and disease using differentially expressed gene data following the treatment of human cells with different perturbagens [28, 29]. CMap contains information on the genetic changes in multiple cell lines after being treated with 19,811 small molecule perturbagens and 314 biological agents, as well as after overexpressing or knocking down 5075 genes [30]. Using this database, we can infer drugs that may cause certain genes to be upregulated or downregulated based on differentially expressed gene data. CMap has already been successfully applied to screen drugs for the treatment of various cancers, including gliomas, pancreatic neuroendocrine tumors, liver cancer, and colon cancer [31–34].

As opposed to single drug treatments, combination therapies involving multiple drugs are increasingly being investigated to treat diseases. Some drugs act synergistically with TMZ to increase the sensitivity of gliomas to it, thus effectively inhibiting glioma proliferation. The proteasome inhibitor bortezomib and TMZ were shown to combine synergistically to significantly decrease cell survival [35, 36]. Afatinib arrests the growth of cancer stem cells (CSCs) and glioma cells by inhibiting the epidermal growth factor receptor variant III-mediated activation of the c-Met and Janus kinase 2/signal transducer and activator of transcription 3 pathways. Moreover, it enhanced TMZ-induced cytotoxicity [37].

In this study, we performed ex vivo experiments to discover that GINS2 regulated the malignant phenotype and TMZ sensitivity of glioma cells, likely by promoting DDR through the early growth response protein 1 (EGR1)/ECT2 axis. The clinical significance of GINS2 was further underscored when we constructed a prognostic model involving the GINS2/EGR1/ECT2 pathways. Using CMap, we screened a GINS2 inhibitor that synergistically inhibited the proliferation of glioma cells along with TMZ. By uncovering a novel mechanism by which GINS2 regulates the sensitivity of glioma to TMZ chemotherapy, these findings provide a promising genetic target for glioma treatment and inform the development of more effective therapeutic approaches.

## Results

### GINS2 is associated with the TMZ-induced DDR and affects the sensitivity of glioma cells to TMZ

GINS2 has been reported to be highly expressed in various tumors [38]. To explore the role of GINS2 in the development of glioma, we examined the mRNA and protein expression of GINS2 in seven glioma cell lines. It was found to be highly expressed in several of them, especially U251, LN229 (Supplementary Fig. 1). Similarly, the expression of GINS2 mRNA was significantly higher in collected glioma samples than paraneoplastic tissues, as evidenced by reverse transcription–quantitative PCR (RT–qPCR) (Supplementary Fig. 2A, B, *P* < 0.05). Bioinformatic analysis of samples from The Cancer Genome Atlas (TCGA) and Genotype-Tissue Expression portal corroborated that GINS2 mRNA was significantly upregulated in tumor tissues compared with normal tissues (Supplementary Fig. 2C, *P* < 0.05). Gliomas classified as grade 1–2 by the World Health Organization are low-grade gliomas (LGGs), while those classified as grade 3–4 are high-grade gliomas (HGGs) [3]. Compared with LGGs, GINS2 expression was higher in HGGs along with higher malignancy (Supplementary Fig. 2D, *P* < 0.05), indicating that GINS2 expression correlated with glioma grade.

**Fig. 1:**
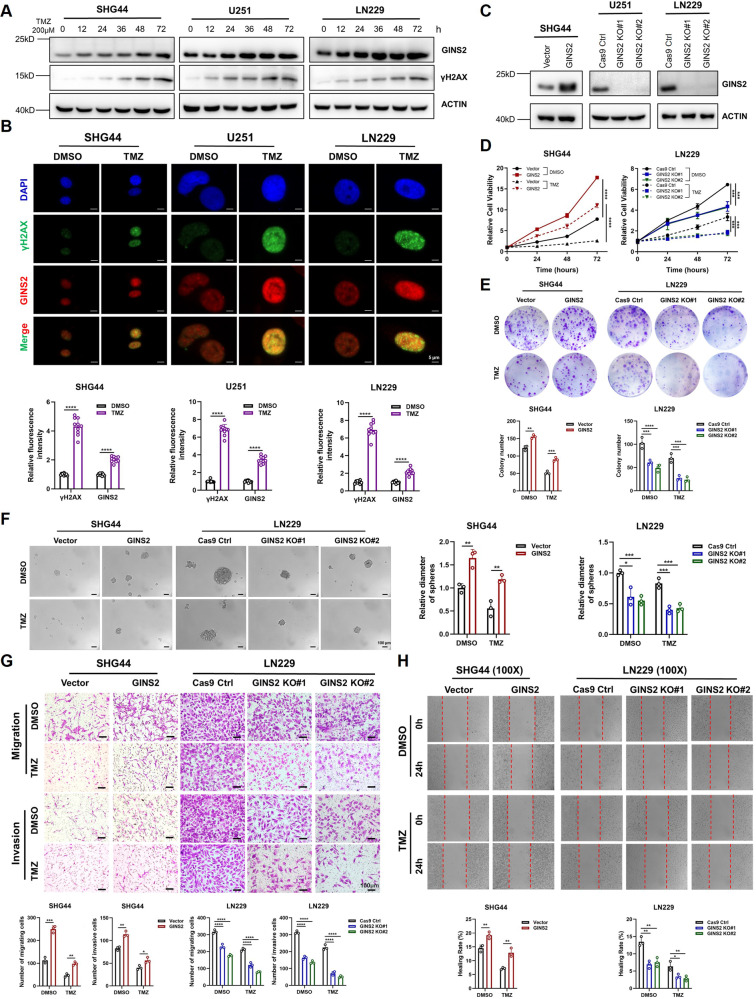
GINS2 is engaged in DDR, promotes malignant phenotype and down-regulates TMZ sensitivity in gliomas. **A** Glioma cells were treated with 200 μM TMZ for 0/12/24/36/48/72 h. Western blottings of GINS2 and γH2AX expression were performed with ACTIN as a control. **B** Immunofluorescence was examined for fluorescence intensity and colocalization of GINS2 and γH2AX in glioma cells after 48 h exposure to 200 μM TMZ. **C** Western blotting verified the construction of GINS2 overexpression and GINS2 knockout cell lines. **D** CCK8 assays were used to examine the effect of GINS2 on glioma cell proliferation and TMZ sensitivity. **E** Colony formation assay to test the colony formation ability of glioma cells with stable GINS2 overexpression/knockout. **F** Stable overexpression or knockout of GINS2 affects glioma cell stemness. **G**, **H** GINS2 promotes the migration and invasive ability of glioma cells. *P < 0.05, **P < 0.01, ***P < 0.001, ****P < 0.0001. *n* = 3 independent experiments. Two-Tailed *t*-Test assuming equal variances. Error bars represent the mean ± standard deviation of the mean.

Using the CCK-8 assay, we determined the half-maximal inhibitory concentration (IC_50_) of TMZ in SHG44 (GINS2 expressed at a relatively low level), U251, and LN229 cells to be 212.3 μM, 394 μM, and 313.1 μM, respectively (Supplementary Fig. 3). To explore the association between TMZ treatment and GINS2 expression, we treated glioma cells with 200 μM TMZ for increasing time durations. γH2AX is a marker of DNA damage and signifies the presence of DDR. GINS2 and γH2AX protein levels were observed to increase with TMZ treatment time (Fig. 1A), indicating that TMZ induced DNA damage and upregulated GINS2 in the cells. The fluorescence intensity of γH2AX and GINS2 increased after TMZ treatment. Moreover, GINS2 and γH2AX partially colocalized, implying that GINS2 may participate in DNA damage repair (Fig. 1B).

To investigate how GINS2 affects the response of glioma cells to TMZ, we engineered SHG44 cells to overexpress GINS2 and knocked it out using clustered regularly interspaced short palindromic repeats (CRISPR)–Cas9 in U251 and LN229 cells, which highly express the protein (Fig. 1C). GINS2 may regulate the sensitivity of glioma cells to TMZ, which in turn also influences the malignant phenotype of cells. Hence, we observed the effects of GINS2 on the proliferation, clone-forming ability, cell stemness, migration and invasion ability, and TMZ sensitivity of glioma cells when treated with TMZ or dimethyl sulfoxide (DMSO). The results of the CCK-8, clone formation, tumor sphere formation, scratch, and Transwell migration and invasion assays showed that GINS2 promoted the malignant phenotype of glioma cells and attenuated their sensitivity to TMZ (Fig. 1D–H, Supplementary Fig. 4).

### GINS2 knockout increases the sensitivity of gliomas to TMZ in nude mice

**Fig. 2:**
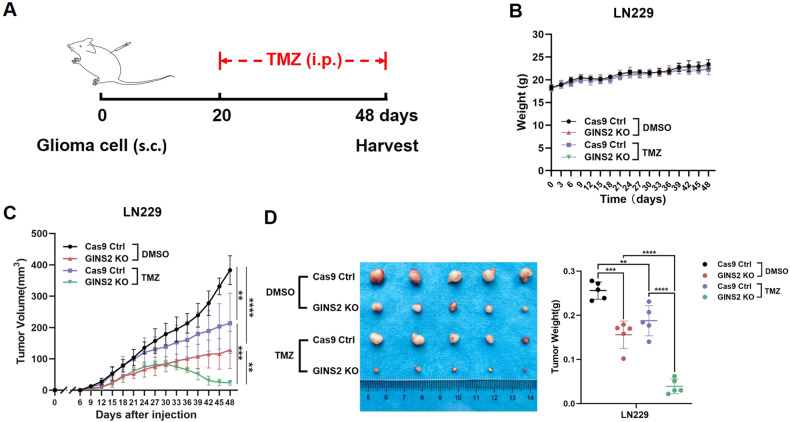
GINS2 affects xenograft glioma proliferation and TMZ sensitivity. **A** Treatment flow diagram of mice. **B** Weight change of mice after inoculating cells. **C** Growth curves of tumor volume in mice. **D** Size and weight of tumors isolated from nude mice. **P < 0.01, ***P < 0.001, ****P < 0.0001. *n* = 5. Two-tailed *t* test assuming equal variances. Error bars represent the mean ± standard deviation of the mean.

We constructed a nude mice xenograft model to investigate how GINS2 regulates TMZ sensitivity in vivo. LN229-GINS2 knockout (KO) cells or LN229-Cas9 control cells were implanted into the subcutis of nude mice, and the growth of the transplanted tumors was monitored. When tumors are more than a volume of about 50 mm^3^, TMZ/DMSO was injected intraperitoneally as the drug treatment (Fig. 2A). Mice body weights were stable and there were no significant growth differences between groups, indicating that none of the different interventions produced significant toxic effects on mice (Fig. 2B). However, the transplanted tumors formed by LN229-GINS2 KO cells were smaller than those in the control group (Fig. 2C), suggesting that depleting GINS2 mitigated the tumor-forming ability of LN229 cells. Compared with the DMSO group, the growth rate of tumors decelerated in the TMZ-treated control group, indicating that TMZ exerted antitumor effects in mice (Fig. 2C). When treated with TMZ, tumors in the Cas9 control group grew at a gradually increasing rate, whereas those in the GINS2 KO group decreased in size. Overall, these results suggest that depleting GINS2 suppressed the tumorigenicity of LN229 cells in vivo and increased the sensitivity of tumors to TMZ (Fig. 2D).

### ECT2 is downstream of GINS2

**Fig. 3:**
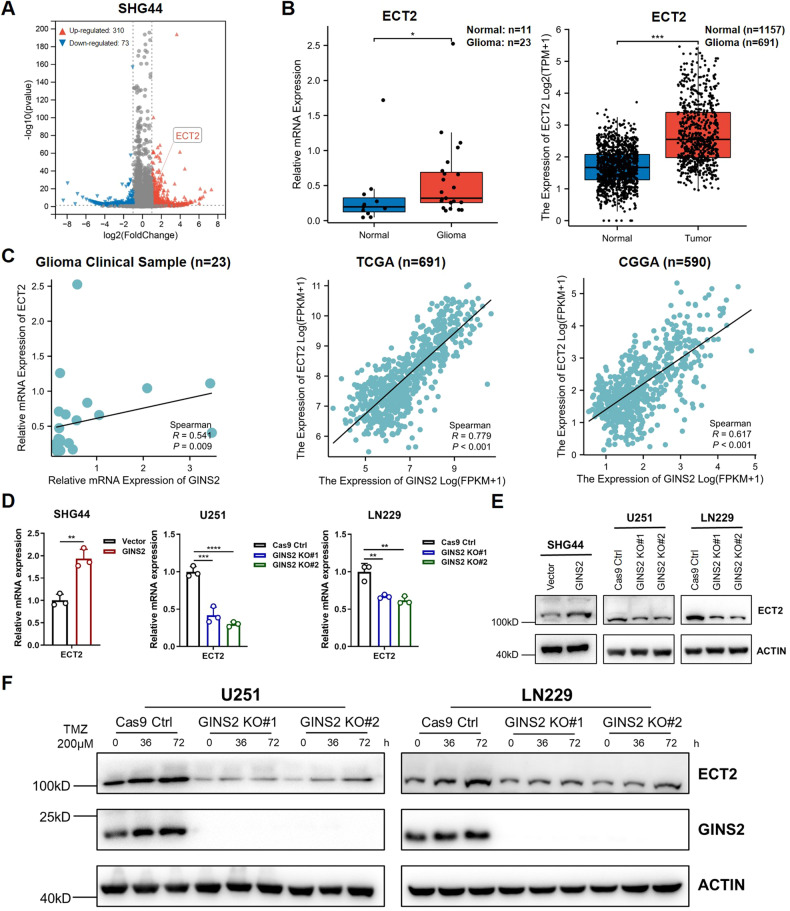
GINS2 regulates the expression of ECT2. **A** Volcano plots present DEGs in SHG44-GINS2 group cells and SHG44-Vector group cells. The red dots on the top right quadrant are significantly upregulated DEGs and the blue dots within the top left quadrant show highly downregulated DEGs in the GINS2 KO compared to SHG44-Vector group; gray dots denote unchanged genes. **B** Comparison of ECT2 expression in normal tissues (left, blue) and in glioma (right, red). In the boxplot, center line as the median, the upper and lower boundaries represent the first and third quartiles, while whiskers extend to 1.5× the interquartile range. **C** Correlation analysis of GINS2 and ECT2 mRNA expression levels. **D** RT-qPCR to detect ECT2 mRNA levels in SHG44-GINS2, and U251/LN229 KO cells. **E** Western blotting analysis of ECT2 in SHG44-GINS2, and U251/LN229 KO cells. **F** Western blotting detecting GINS2 and ECT2 in TMZ (200 μM) treated U251 KO cells and LN229 KO cells. *P < 0.05, **P < 0.01, ***P < 0.001, ****P < 0.0001. *n* = 3 independent experiments. Two-Tailed *t* test assuming equal variances. Error bars represent the mean ± standard deviation of the mean.

The mechanism by which GINS2 regulates the malignant phenotype and TMZ sensitivity of glioma is unclear. With the criteria |fold change| > 2 and *P* < 0.05, comparative RNA sequencing of SHG44-GINS2 and SHG44-Vector cells revealed 310 upregulated and 73 downregulated genes in the former (Fig. 3A). We analyzed the expression correlation of all genes with GINS2 based on TCGA-Glioma (*n* = 691) and CGGA-Glioma (*n* = 590), and obtained 411 and 203 genes positively correlated with GINS2 expression (*R* > 0.6, *P* < 0.05), respectively, and intersected them with 310 up-regulated genes obtained from RNA sequencing, with 34 intersected genes being presented (Supplementary Fig. 5). We ranked 34 genes from smallest to largest *P*-value in RNA sequencing results, and ECT2 was ranked as the first. ECT2 is an important regulator of DNA double-strand break repair and genome stability [26, 27]. The expression of ECT2, was also verified by RT–qPCR in 11 clinical and 23 glioma samples, wherein it was significantly upregulated compared with normal tissues (Fig. 3B, *P* < 0.05). Bioinformatic analysis yielded the same results (Fig. 3B, *P* < 0.001). In addition, the mRNA expressions of GINS2 and ECT2 were positively correlated in glioma (Fig. 3C). Using RT-qPCR and Western blotting, we demonstrated that GINS2 positively regulated the expression of ECT2 (Fig. 3D, E). Furthermore, although treating GINS2 KO cells with 200 μM TMZ upregulated ECT2, the protein was still lower than that of Cas9 Ctrl group under the same treatment conditions (Fig. 3F), indicating that ECT2 may be largely regulated by GINS2 under TMZ treatment.

### GINS2 regulates the malignant phenotype and TMZ sensitivity of glioma via ECT2

**Fig. 4:**
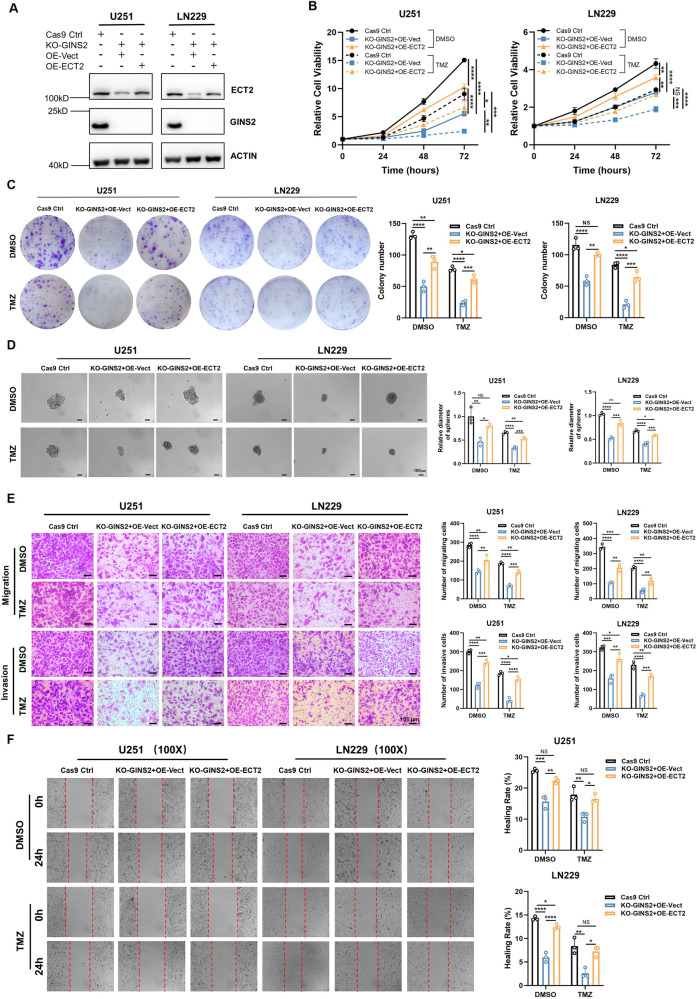
ECT2 mediates the regulation of GINS2 on malignant phenotype and TMZ sensitivity in glioma cells. **A** Western blotting to detect the effect of transferring the ECT2 plasmid in GINS2 knockout cells. **B** Cell proliferation measurement of Cas9 Ctrl, KO-GINS2 + OE-Vector and KO-GINS2 + OE-ECT2 glioma cells. **C** Clone-formation ability measurement of Cas9 Ctrl, KO-GINS2 + OE-Vector and KO-GINS2 + OE-ECT2 glioma cells. **D** Stemness measurement of Cas9 Ctrl, KO-GINS2 + OE-Vector and KO-GINS2 + OE-ECT2 glioma cells. Invasion and migration capacity measurement (**E**, **F**) of Cas9 Ctrl, KO-GINS2 + OE-Vector and KO-GINS2 + OE-ECT2 glioma cells. NS: No significance. *P < 0.05, **P < 0.01, ***P < 0.001, ****P < 0.0001. *n* = 3 independent experiments. Two-tailed *t*-test assuming equal variances. Error bars represent the mean ± standard deviation of the mean.

To determine whether GINS2’s regulation of the malignant phenotype and TMZ sensitivity of glioma was mediated by ECT2, we overexpressed ECT2 in GINS2 KO cells for rescue experiments. Western blotting confirmed the rescue of ECT2 expression in GINS2 KO cells (Fig. 4A). The CCK-8 and colony formation assays showed that upregulating ECT2 reversed the GINS2 KO-mediated decrease in the proliferative capacity and colony formation ability of glioma cells (Fig. 4B, C). Tumor sphere formation assays demonstrated that the partial stemness of GINS2 KO cells was restored when ECT2 was overexpressed (Fig. 4D). Transwell migration and invasion assays and scratch assays confirmed that the migration and invasion of GINS2 KO cells increased when ECT2 was overexpressed (Fig. 4E, F). Therefore, the upregulation of ECT2 reversed the GINS2 KO-mediated reduction in proliferation, stemness, migration, invasion, and TMZ resistance of glioma cells. These findings confirm that GINS2 regulates the malignant phenotype and TMZ sensitivity of gliomas via ECT2.

### GINS2 modulates ECT2 expression by affecting the stability of EGR1 mRNA

**Fig. 5:**
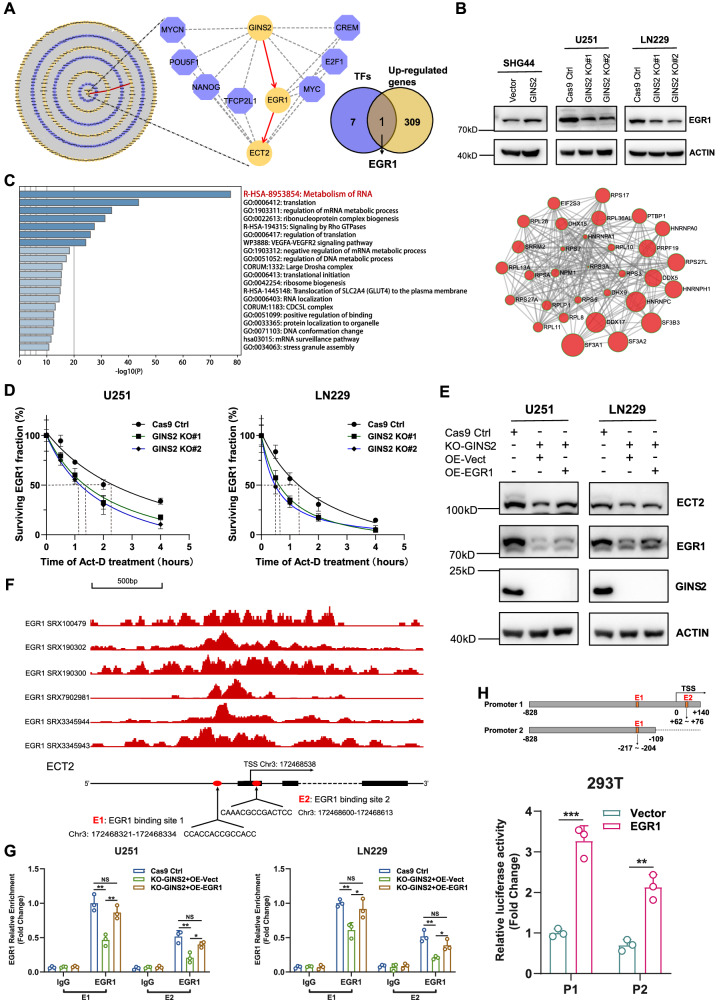
GINS2 regulates ECT2 transcription levels through TF EGR1. **A** A transcription factor regulatory network of upregulated genes in SHG44-GINS2 cells was constructed. Venn diagram showed the intersection of transcription factors (purple) and differentially up-regulated genes (yellow) in regulatory network. **B** Western blotting to detect the regulatory effect of GINS2 on EGR1. **C** Enrichment analysis of GINS2-binding proteins. Protein and protein interaction network of GINS2-bind RNA metabolism-related proteins. **D** mRNA stability assay to assess the effect of GINS2 on the mRNA stability of EGR1. **E** Western blotting was performed to detect changes in GINS2, EGR1 and ECT2 protein expression after the transfer of EGR1 plasmids in GINS2 knockout glioma cells. **F** Database prediction of EGR1 binding sites in the ECT2 promoter region. **G** ChIP assay to explore the enrichment of EGR1 in the promoter region of ECT2 gene in GINS2 knockout cells overexpressing EGR1 and control cells. **H** Schematic diagram of the dual luciferase reporter gene truncator construct. Dual luciferase reporter gene experiments further confirmed the positive regulation of EGR1 as a transcription factor on ECT2. Act-D Actinomycin D, TSS Transcription start site, E1 EGR1 binding site 1, E2 EGR1 binding site 2, P1 Promoter 1, P2 Promoter 2, NS No significance. *P < 0.05, **P < 0.01, ***P < 0.001. *n* = 3 independent experiments. Two-tailed *t* test assuming equal variances. Error bars represent the mean ± standard deviation of the mean.

GINS2 may regulate the mRNA expression of ECT2 via transcription factors (TFs). Therefore, we constructed a regulatory network of TFs for the 310 upregulated genes in SHG44-GINS2 cells, and identified eight possible TFs in the pathway starting with GINS2 and ending with ECT2. Only one of the eight was upregulated in the sequencing results, which was *EGR1* (Fig. 5A). We hypothesized that *EGR1* was a downstream target of GINS2 and speculated about the existence of a GINS2–EGR1–ECT2 regulatory pathway. Western blotting showed that EGR1 protein levels were elevated after GINS2 was overexpressed and decreased after GINS2 was knocked out, verifying the positive regulatory effect of GINS2 on EGR1 (Fig. 5B).

To identify the proteins that interact with GINS2 inside the cell, we pulled down endogenous GINS2 from the GINS2-high-expressing U251 cell line using an anti-GINS2 antibody followed by liquid chromatography–tandem mass spectrometry. Functional enrichment analysis revealed that the pulled-down proteins were mainly associated with the regulation of RNA metabolism, so we plotted an interaction network of RNA metabolism-related proteins (Fig. 5C). Using RT–qPCR, we detected the mRNA expression of EGR1 in GINS2 KO and control cells after treating them with 5 μg/mL actinomycin D. The half-life of EGR1 mRNA was shorter in U251/LN229 GINS2 KO cells (Fig. 5D), indicating that GINS2 modulated the stability of EGR1 mRNA. In addition, our bioinformatics predictions suggest that among the 10 RNA metabolism-associated proteins interacting with GINS2, DHX9 is likely to have the highest probability of binding to the EGR1 mRNA (Supplementary Tables 3, 4).

To determine whether GINS2 regulated ECT2 expression via EGR1, we transfected GINS2 KO cell lines with EGR1-encoding plasmids. Western blotting showed that the expression of ECT2 increased after EGR1 was overexpressed, which demonstrates that GINS2 regulated ECT2 expression through EGR1 (Fig. 5E). Then, we used JASPAR to predict EGR1 binding sites in the promoter region of ECT2, followed by ChIP-Atlas to present the binding peak map of EGR1 base on the chromatin immunoprecipitation-sequencing dataset (Fig. 5F), indicating that EGR1 may initiate transcription from multiple regions in the ECT2 promoter, with the E1 and E2 sites carrying the highest probability. Chromatin immunoprecipitation (ChIP) showed that EGR1 could bind to both of these sites. Moreover, enrichment of EGR1 in the promoter region of the ECT2 gene decreased after GINS2 was knocked out but increased after EGR1 was overexpressed (Fig. 5G, *P* < 0.05). Dual luciferase reporter gene assays also showed that luciferase activity was stronger when EGR1 was overexpressed compared with the control group, indicating that EGR1 acts as a TF to positively regulate the transcription of ECT2 through the E1 and E2 sites (Fig. 5H).

### The clinical significance of the GINS2–EGR1–ECT2 pathway

**Fig. 6:**
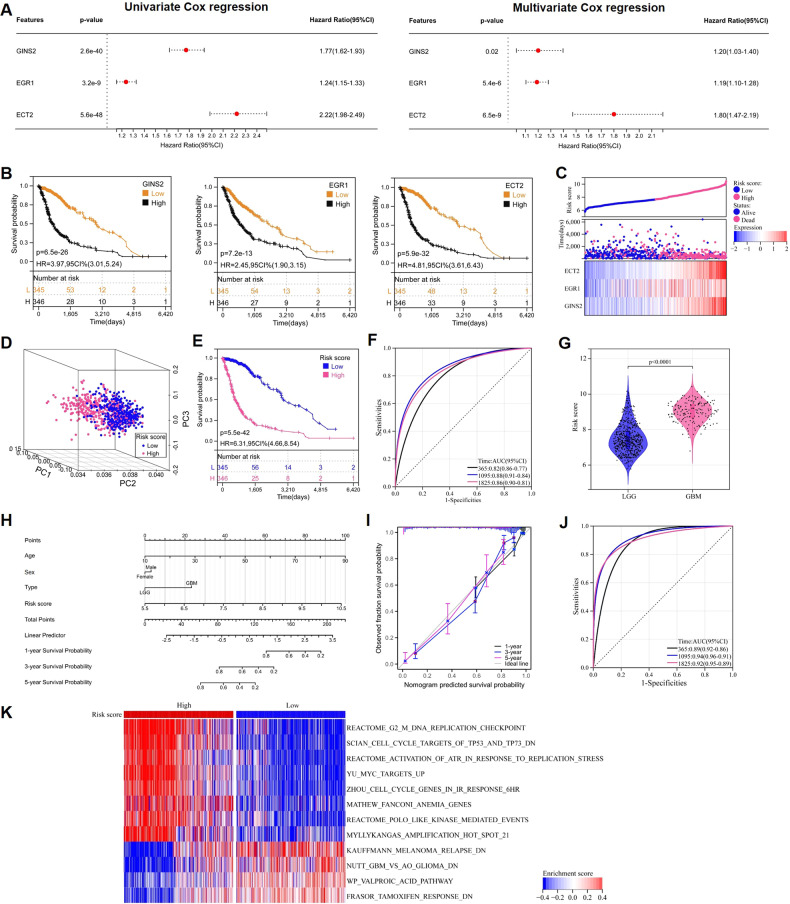
Construction and validation of GEEPS. **A** Univariate Cox and multivariate Cox regression analyses were performed for GINS2, EGR1, and ECT2, respectively. **B** K-M curve to analyze the relationship between GINS2/EGR1/ECT2 and OS of glioma patients, respectively. **C** Heat map of GINS2, EGR1 and ECT2 gene expression, risk score curve and scatter plot of survival status in glioma patients. **D** PCA analysis to determine the clustering performance of GEEPS. **E** K-M analysis of patients with glioma in the high-risk and low-risk groups. **F** AUC of ROC curves were used to validate GEEPS’s accuracy in predicting glioma patients’ 1-, 3-, and 5-year survival. **G** Comparison of risk scores for LGG and HGG patients. In the violin plots, center line as the median, the upper and lower boundaries represent the first and third quartiles, while whiskers extend to 1.5× the interquartile range. **H** A Nomogram was constructed to predict 1-, 3-, and 5-year survival of glioma patients based on four independent prognostic factors (risk score, age, sex and glioma grade typing). **I** Calibration plots to verify the accuracy of the Nomogram. **J** ROC curve analysis of the Nomogram. **K** GSVA analysis of activation pathways in the high-risk and low-risk groups. GEEPS GINS2-EGR1-ECT2 pathway signature, OS Overall survival, PCA Principal component analysis, K-M Kaplan-Meier, ROC Receiver operating characteristic curve, AUC Area under the curve, LGG Low-grade glioma, GBM Glioblastoma. Two-tailed *t* test assuming equal variances. Error bars represent the mean ± standard deviation of the mean.

Having identified the GINS2–EGR1–ECT2 pathway, we performed further bioinformatic analysis on these three genes to explore their prognostic significance. Firstly, a univariate Cox regression analysis of these genes based on the glioma samples from TCGA (*n* = 691) revealed a hazard ratio greater than 1 for all three of them (Fig. 6A), reflecting their association with patient survival. Multifactorial Cox regression analysis further indicated that these genes were independent prognostic indicators (Fig. 6A). Kaplan–Meier (K–M) survival curves validated that the upregulation of these three genes was associated with poor prognosis of glioma patients (Fig. 6B).

Based on multifactorial Cox regression coefficients and the expression of the three genes, we constructed a prognostic model that we named GINS2–EGR1–ECT2 pathway signature or GEEPS. The risk value for each patient was calculated as follows: risk score = GINS2 × 0.18 + EGR1 × 0.17 + ECT2 × 0.58. The glioma samples from TCGA (*n* = 691) were classified into high- and low-risk groups according to the median value of the risk score. Heat maps of the risk score, survival status, and the expression of the three genes showed that the high-risk group had higher prognostic gene expression and number of deaths than the low-risk group (Fig. 6C). Principal component analysis (PCA) verified that this strategy of stratification divided the glioma samples from TCGA into two independent categories (Fig. 6D). K–M survival analysis showed that patients in the high-risk group had significantly shorter overall survival (OS) than those in the low-risk group (*P* < 0.05, Fig. 6E). The area under the curve (AUC) values of the receiver operating characteristic (ROC) curve of this model to predict patient survival at 1, 3, and 5 years were 0.82, 0.8, and 0.86, respectively (Fig. 6F), indicating the sensitivity and specificity of this model for predicting the survival of glioma patients. In addition, we analyzed the association between the GEEPS risk score and the malignancy of glioma. GBM had a higher risk score than LGGs with a lower malignancy, which is consistent with the clinical results (Fig. 6G). To visualize GEEPS, we constructed a nomogram by combining the risk scores, age, sex, and tumor grade of glioma patients from the TCGA dataset to predict the 1-, 3-, and 5-year survival for each patient (Fig. 6H). The calibration plot of each fitted line almost overlapped with the ideal curve, reflecting the accuracy of the nomogram prediction (Fig. 6I). ROC curve analysis showed that the AUC values corresponding to 1-, 3-, and 5-year survival were about 0.9, demonstrating the sensitivity and specificity of this nomogram for predicting the survival of patients (Fig. 6J). The independent prognostic value and broad applicability of GEEPS were further demonstrated by analyzing a Chinese Glioma Genome Atlas (CGGA) dataset (*n* = 590) as an external cohort (Supplementary Fig. 6).

The high- and low-risk subgroups of the glioma samples from TCGA were subjected to Gene Set Variation Analysis (GSVA) to explore the pathways enriched in this model. The pathway heat map showed that DDR-related pathways were enriched in the high-risk group (Fig. 6K). p53 plays a crucial role in response to DDR, and despite DNA damage, downregulated p53 leads to continued cell proliferation, which fuels malignant transformation [39]. In response to replication stress, the ataxia telangiectasia and Rad3-related protein pathway were also activated in the high-risk group. Therefore, tumor cells in the high-risk group of patients exhibit a more robust capacity to repair DNA damage and develop resistance to TMZ chemotherapy. As a result, gliomas with a higher risk score may be less sensitive to TMZ, suggesting that GEEPS can be a good predictor of TMZ sensitivity in glioma patients and can guide clinical treatment strategies to some extent.

### Pal/BIX-02189 dampens GINS2 expression and synergistically inhibits glioma cell proliferation, stemness, invasion and migration capacity with TMZ

**Fig. 7:**
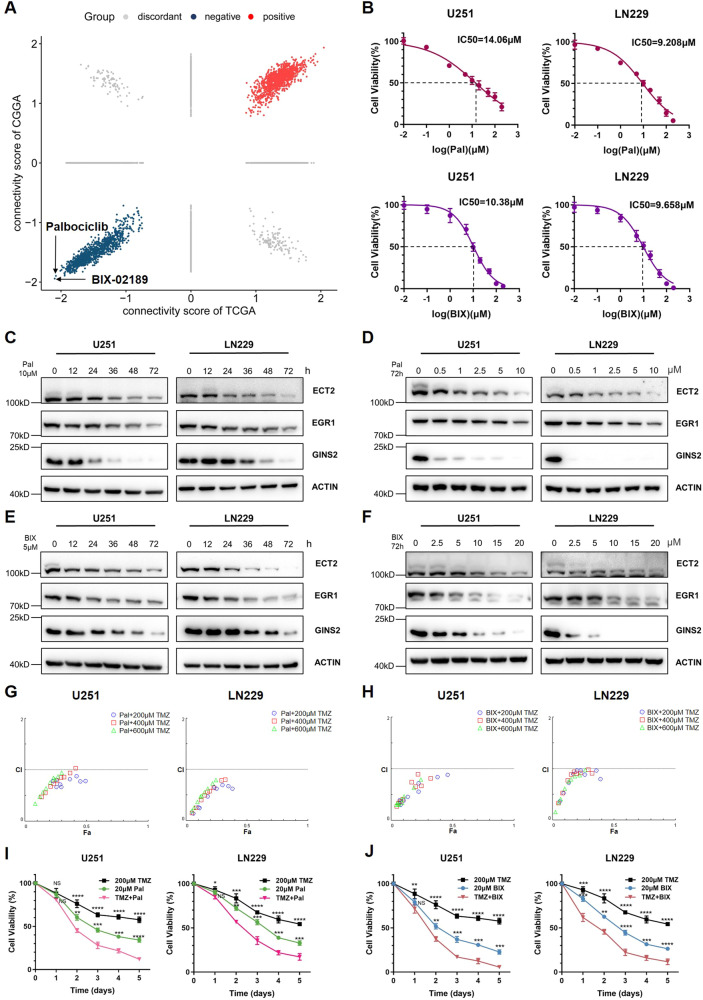
Exploration of the combined effect of GINS2 inhibitor and TMZ. **A** Two potential inhibitors of GINS2 were screened by CMap database. **B** CCK8 assay to detect the IC_50_ of Pal, BIX-02189 in glioma cell lines U251 and LN229. **C** U251 and LN229 were treated with 10 μM Pal time gradient and Western blotting detected protein levels of GINS2, EGR1 and ECT2. **D** Concentration gradient of Pal treated U251 and LN229 for 72 h. Western blotting assays were performed to detect the protein levels of GINS2, EGR1 and ECT2. **E** U251 and LN229 were treated with 5 μM BIX time gradient and Western blotting detected protein levels of GINS2, EGR1 and ECT2. **F** A concentration gradient of BIX treated U251 and LN229 for 72 h. Western blotting assays were performed to detect the protein levels of GINS2, EGR1 and ECT2. **G** U251/LN229 were treated with different concentrations of Pal in combination with different concentrations of TMZ, and CCK8 was used to assess cell viability and CompuSyn software was used to calculate CI. **H** U251/LN229 cells were treated with different concentrations of BIX in combination with different concentrations of TMZ, and CCK8 was used to assess cell viability and CompuSyn software was used to calculate CI. **I** CCK8 experiments to assess cell viability after treatment of U251/LN229 with TMZ alone, Pal or a combination of the two drugs in a time gradient. **J** Cell viability was assessed in CCK8 assays after treatment of U251/LN229 with TMZ alone, BIX-02189 or a two-drug combination time gradient. Pal Palbociclib, BIX BIX-02189, CI Combination index, NS No significance. *P < 0.05, **P < 0.01, ***P < 0.001, ****P < 0.0001. *n* = 3 independent experiments. Two-tailed *t* test assuming equal variances. Error bars represent the mean ± standard deviation of the mean.

GINS2 inhibitors may effectively suppress glioma cell proliferation by enhancing the TMZ sensitivity of the cells. Since GINS2-related inhibitors have not yet been reported, we screened for them using CMap. More minor scores indicate more significant inhibition of GINS2 mRNA expression. We predicted two GINS2 inhibitors with the most minor scores in both the TCGA and Chinese Glioma Genome Atlas databases: Palbociclib (Pal) and BIX-02189 (Fig. 7A). Using the CCK-8 assay, we determined that the IC_50_ of Pal in U251 and LN229 cells was 14.06 μM and 9.208 μM, respectively, while that of BIX-02189 was 10.38 μM and 9.658 μM, respectively (Fig. 7B). U251 and LN229 cells were treated with Pal/BIX-02189 at different dosages for different durations, we found that both drugs significantly inhibited the protein levels of GINS2, EGR1, and ECT2 (Fig. 7C–F).

Next, we investigated the combined effect of Pal/BIX-02189 and TMZ. U251 and LN229 cells were treated with different concentrations of Pal/BIX-02189 (0, 2.5, 5, 10, 15, 20, 25, 30, 40, or 50 μM) or/and TMZ (200, 400, or 600 μM) for 72 h. The combination indexes thus obtained were less than 1 (Fig. 7G–H, Supplementary Tables 6, 9), indicating that the combination of Pal/BIX-02189 and TMZ synergistically inhibited the proliferation of glioma cells. In addition, we used the CCK-8 assay to assess the effect of the GINS2 inhibitor–TMZ combination at different time points. The combination exerted a more significant antiproliferative effect than a single drug under multiple treatment conditions (Fig. 7I–J). Collectively, Pal/BIX and TMZ combination inhibit proliferation, stemness, invasion and migration capacity of glioma cells (Supplementary Fig. 7).

**Fig. 8:**
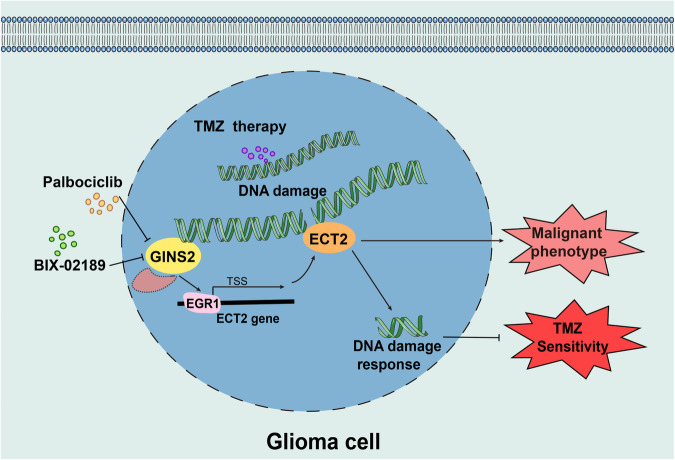
Model for this study. GINS2 regulates TMZ chemosensitivity of gliomas by participating in DDR through the EGR1/ECT2 axis, and GINS2 inhibitor (Palbociclib /BIX-02189) and TMZ synergistically inhibit glioma cell proliferation, stemness, invasion and migration capacity.

In light of these findings, we illustrated a graphical abstract (Fig. 8) showcasing the regulatory role of GINS2 in TMZ chemosensitivity through the EGR1/ECT2 axis in glioma cells.

## Discussion

Glioma cells treated with TMZ may be directly fated for programmed cell death, but may also strive for a slim chance of survival by orchestrating the cell cycle and DDR, which require the combined action of multiple proteins. γH2AX, the Ser139-phosphorylated form of histone H2AX, marks the cell’s response to DSBs [40]. The elevated levels of γH2AX observed in the nuclei of TMZ-treated glioma cells suggest that TMZ-induced DNA alkylation in these cells is not successfully repaired by pathways like the O^6^-methylguanine-DNA methyltransferase pathway and mismatch repair, but instead progresses to the final step of DNA damage: DSBs. The elevated expression of GINS2 and its partial colocalization with γH2AX after TMZ treatment suggest that GINS2 responds positively to DNA damage and may be involved in DSB repair.

This study demonstrates that GINS2 affected the proliferation, clone formation, stemness, migration, and invasion of glioma cells, as reported in other cancers as well [38]. In addition, we discovered for the first time that GINS2 regulated drug sensitivity in tumor cells. Deficiency of one or more CMG helicase-related genes may lead to DNA replication stress and damage [41]. Fan Xuan et al. showed that the downregulation of MYC-induced nuclear antigen decreased the expression of various CMG helicase-related genes, including GINS2, induced DNA damage, and increased the sensitivity of GBM cells to the DNA-toxic drug doxorubicin [42]. In this context, reserve GINS2 may facilitate genomic stability under replicative stress. In contrast, GINS2 KO could be devastating for the function of the GINS complex or even the CMG helicase. GINS2 may regulate the TMZ sensitivity of glioma via its downstream targets, such as AKT [15, 43], ataxia telangiectasia mutated, and checkpoint kinase 2 [21]. Furthermore, the drug sensitivity of tumor cells is associated with malignant phenotypes. For example, CSCs in GBM are inherently resistant to TMZ treatment [44], so GINS2 may regulate TMZ sensitivity through pathways other than the DDR. More experiments need to be performed to prove these speculations.

ECT2 is upregulated in several human cancers and functions as an oncogene [45–47]. ECT2 promoted glioma cell proliferation by regulating the expression of the deubiquitinating enzyme PSMD14 to affect the degradation of the TF E2F1 [48]. ECT2-associated functional rescue experiments in this study also demonstrated that ECT2 promotes the proliferation, clone formation, cell stemness, migration, and invasion of glioma cells. By participating in the DDR [26, 27], it may also regulate the TMZ sensitivity of glioma cells, as evidenced by functional rescue experiments. Notably, ECT2 was strongly associated with the classical tumor suppressor gene p53. p53 is required for apoptosis and the activation of the S and G_2_/M checkpoints during DDR. In gastric cancer cells treated without inducing factors, wild-type p53 protein downregulated ECT2 whereas mutant p53 upregulated it, promoting the proliferation, migration, and invasion of gastric cancer cells [49]. The potential regulatory role of p53 on ECT2 may be beneficial in explaining ECT2 expression levels were also affected by TMZ treatment in the case of GINS2 knockout. More interestingly, the oncogenic role of ECT2 is reflected in the manner in which it regulates p53. In the early stages of DNA damage in primary mouse embryonic fibroblasts, ECT2 was found localized at foci of chromosomal damage, promoting p53 phosphorylation and orchestrating cell cycle arrest, but in the late stages, ECT2 was degraded. This whole process reflects the dynamic response of ECT2 to DNA damage [50]. Besides regulating p53, ECT2 deficiency leads to the sustained activation of poly (ADP-ribose) polymerase 1 and facilitates the repair of ribosomal DNA damage, promoting radiotherapy resistance in lung and nasopharyngeal carcinoma cells [51]. Thus, ECT2 may play a dual role in DNA damage repair depending on the cell type, growth and stress conditions, and even the status of p53.

RNA-binding proteins affect pre-mRNA splicing and the stability, localization, editing, and translation of specific RNAs [52]. Mass spectrometry analysis revealed that most proteins bound to GINS2 regulated RNA metabolism. RNA stability experiments demonstrated that GINS2 KO shortened the half-life of EGR1 mRNA, suggesting that GINS2 may bind to and interact with one or more RNA-binding proteins to influence the mRNA stability of EGR1. However, more experiments are needed to elucidate the specific regulatory processes.

EGR1 serves as a TF in cell proliferation, differentiation, apoptosis, and several other processes [53]. In glioma, EGR1 induced the methyltransferase METTL3 to promote the proliferation and self-renewal of CSCs [54]. Moreover, EGR1 downregulation induced transcriptional repression, which may help Furanodienone, a potential anticancer drug, to overcome TMZ resistance in GBM [55]. In normal neurons, etoposide-induced DSBs stimulated EGR1 expression, which may be a response to DNA damage [56]. Moreover, etoposide-induced BRCA1 promoter activity in cervical cancer cells was mediated by upregulated EGR1, which confirms the link between EGR1 and DNA damage repair [57].

The mechanisms responsible for the high expression of ECT2 in various tumor tissues are unknown, and no study has investigated the TFs that regulate ECT2. We predicted and validated EGR1 to be a TF that controls ECT2 expression. Further, GINS2 KO inhibited the transcriptional regulation of ECT2 by EGR1, demonstrating the existence of the GINS2–EGR1–ECT2 regulatory pathway. Prognostic models employ various predictors to assess the risk of disease progression in patients. In this study, we constructed the GEEPS, which independently predicted the prognosis of glioma patients. Its relationship with DDR was also consistent with previous experimental results. These findings highlight the clinical significance of the GINS2–EGR1–ECT2 pathway.

Pal, a cyclin-dependent kinase 4/6 (CDK4/6) inhibitor, is often used to treat advanced estrogen receptor-positive, human epidermal growth factor receptor 2-negative breast cancer [58]. Several studies have explored the possibility of using Pal to treat glioma. For example, Pal combined with radiotherapy significantly prolonged the survival of GBM mice compared with monotherapy [59]. Another in vitro study showed that using Pal with or after radiotherapy inhibited DSB repair and promoted apoptosis in GBM cells [60]. Zhenzhe Li et al. revealed that the Pal–TMZ combination downregulated CDK6 and reduced M2 polarization in microglia, thereby inhibiting the growth of TMZ-resistant glioma cells [61]. The downregulation of GINS2 in pancreatic cancer cells resulted in a significant downregulation of CDK4/6. Furthermore, CDK inhibited the loading of the MCM complex by phosphorylating the origin recognition complex and promoting the interaction between cell division cycle protein 45, MCM, and GINS proteins [62, 63]. Consequently, a deeper crosstalk may also exist between CDK4/6 or the CDK family and GINS2.

BIX-02189 has been less studied than Pal, and is not yet in clinical practice. Being an inhibitor of mitogen-activated protein kinase kinase 5/extracellular signal-regulated kinase 5, BIX-02189 has been reported to inhibit diffuse glioma cell growth and promote apoptosis [64, 65], but no studies have explored the effect of this drug in combination with TMZ. In the present study, we identified Pal/BIX-02189 as GINS2 inhibitors through the CMap database and experiments. Inhibitors were screened from the CMap database based on changes in gene expression, which is distinct from the criterion for screening small molecule inhibitors (small organic molecules that reduce protein activity or impede biochemical reactions) that target the protein of interest. The specificity and potential mechanisms of GINS2 inhibition by these drugs warrant follow-up studies. In addition, Pal/BIX-02189 and TMZ synergistically inhibited glioma cell proliferation, at least in part by inhibiting GINS2 and DDR.

In conclusion, GINS2 regulated the TMZ sensitivity of glioma through the EGR1/ECT2 axis as well as the DDR pathway, and the GINS2–EGR1–ECT2 pathway has clinical implications for predicting the prognosis of glioma patients. Pal and BIX-02189 are novel inhibitors of GINS2 that synergistically inhibited glioma proliferation with TMZ. These findings reveal a novel mechanism by which GINS2 regulates the chemosensitivity of glioma to TMZ, and are expected to provide a promising genetic target for glioma treatment while guiding the development of more effective combination therapies.

## Methods

### Cell culture and transfection

Glioma cell lines U373 (ATCC: HTB-17), A172 (ATCC: CRL-1620), LN229 (ATCC: CRL-2611), U87(ATCC: HTB-14), and T98G (ATCC: CRL-1690) were purchased from American Type Culture Collection (ATCC). Glioma cell lines SHG44, U251 and human embryonic kidney 293 T cells were gifts from the Cancer Research Institute of Central South University. All cells were maintained in DMEM (Gibco, Carlsbad, CA, USA), supplemented with 10% fetal bovine serum (FBS, Biological Industries, Israel), 100 mg/mL penicillin, and streptomycin solution, at 37 °C with 5% CO_2_. All cell lines tested negative for mycoplasma contamination and were passaged <10 times after initial recovery from the frozen stocks.

The GINS2 plasmid (CH8926291, WZ Biosciences, Jinan, China) was generated by inserting the GINS2 CDS region into the plvx-EF1a-puro vector. The single guide RNA (sgRNA) plasmid targeting human GINS2 and control construct were purchased from General Biol (Anhui, China), with cloning vector being lentiCRISPR v2. The sgRNA target sequence used is shown in Supplementary Table 1. pLV2-CMV-ECT2-Neo plasmid (P38188), pLVX-CMV-EGR1-Neo plasmid (P237880) and corresponding vector plasmid were purchased from MiaoLing Biologicals (Wuhan, China). For the lentiviral infection assay, steps were performed as previously described [66].

### RT-qPCR

Detailed information about RT-qPCR assay was previously described [66]. Trizol reagent (R401-01, Vazyme) was used for RNA extraction, and HiScript ® II Q RT SuperMix for qPCR (+gDNA wiper) (R223-01, Vazyme) was applied for reverse transcription of RNA. Real-time quantitative PCR was performed by Bio-Rad CFX Connect Real-Time PCR System and MonAmp™ ChemoHS qPCR Mix (MQ00401S, Monad). All primer sequences are shown in Supplementary Table 2.

### Western blotting

Details regarding the Western blotting have been previously described [67]. The primary antibodies for GINS2 (#16247-1-AP, 1:4000) and EGR1(#22008-1-AP, 1:1000) were purchased from Proteintech, and the Anti-ECT2 antibody (#A20389, 1:1000) was purchased from Abclonal. Anti-γH2AX antibody (#M63324S, 1:2000) was purchased from Abmart. The primary antibody against β-actin was purchased from Affinity (#T0022, 1:4000). HRP-conjugated corresponding secondary antibodies are Goat Anti-Rabbit IgG (H + L) HRP (S0001, Affinity) and Goat Anti-Mouse IgG (H + L) HRP (S0002, Affinity). Unprocessed blots are provided in Uncropped Western Blots.

### Cell immunofluorescence

Cells affixed to slides were incubated in a fresh medium containing 200 μM TMZ/DMSO for 48 h. After three washes with PBS, cells were fixed with 4% paraformaldehyde and then left in 0.2% Triton for 10 min to disrupt the cell membrane. 200 μL of blocking solution was added to each well and the cells were treated for 1 h at room temperature. Anti-GINS2 antibody (Proteintech, #16247-1-AP, 1:200) and anti-γH2AX antibody (Abmart, #M63324S, 1:200) was used to culture the cells overnight at 4 °C. Goat Anti-Rabbit IgG (Dylight 594, Abbkine, A23420, 1:200) and Goat Anti-Mouse IgG (DyLight 488, Abbkine, A23210, 1:200) were used to conjugate the corresponding primary antibodies for 1 h. After washing again, the nuclei were stained with DAPI for 30 min and sealed with neutral gum. Fluorescence and colocalization were observed with laser scanning confocal microscopy (Leica, TCS SP8 SR).

### Cell counting Kit-8 (CCK8) assay

Live cells were measured using the CCK-8(A311-01, Vazyme) as described previously [68, 69]. Glioma cell lines were inoculated at 2000 cells per well in 96-well plates and incubated for 24 h to allow cell attachment. Various concentrations of inhibitors were added to the indicated wells and the cells were incubated for 72 h. The original medium is discarded and 100 µL of a 9:1 mixture of medium and CCK8 reagent is added to each well and incubated at 37 °C for 2 h. The absorbance of each sample is then measured at 450 nm using a microplate reader (Biotek, Synergy 2). IC_50_ values are calculated using GraphPad Prism 8.

The cell proliferation assay was performed by inoculating glioma cell lines with 2000 cells per well in 96-well plates for 24 h. The next day, cells were treated with 200 µM TMZ (Selleck, S1237) or DMSO for 0/24/48/72 h. Cell viability assays were performed as described above.

For combined drug effect analysis, glioma cells U251/LN229 were inoculated overnight at 2000 cells per well in 96-well plates and then were treated with Palbociclib (P835899, Macklin)/BIX-02189 (A5801, Apexbio), TMZ or control vehicle for 72 h, as single agents as well as in combination. CCK-8 was used to detect cell viability and combination index (CI) plots were created by using CompuSyn software (Cambridge, UK) to quantify drug combination effects. Drug synergism, additive and antagonistic effects were defined by CI values of <1, =1 and >1, respectively.

### Colony-forming assay

Approximately 500 Glioma cells were seeded into 6-well plates and cultured for 24 h, and then cells were treated with either DMSO, 200 μM TMZ for 12 days. Cells were fixed in methanol for 15 min and stained with 0.1% crystal violet for 20 min. Microscopy and ImageJ software to visualize and count the number of clones.

### Oncosphere formation assay

The cells to be tested were washed twice with serum-free and antibiotic-free DMEM/F12. A dedicated medium containing 1×B27(12587010, Thermo Fisher), EGF (20 ng/ml final concentration, PRP100159, AbbKine), Bfgf (20 ng/ml final concentration, PRP1010, AbbKine), 200 μM TMZ/DMSO was added in a low adherence 12-well plate (Corning, NY, USA) and inoculated with 1000 cells per well. Cells were cultured for 7–14 days and the cell-forming state should be observed and photographed.

### Cell scratch test

Gliomas were inoculated into six-well plates at 2.5 × 10^5^ cells per well. After 24 h, the full-grown cells were evenly scraped across with a 200 μL pipette tip, and the medium was replaced with FBS-free but 200 μM TMZ/DMSO-containing medium. Photographs were taken to record the scratch areas at 0 h and 24 h of incubation. The healing area was analyzed using ImageJ.

### Transwell assay

Previous studies have described the relevant details of the Transwell assay [69].

### Tumor xenograft assays in nude mice

The SCID mice (Female, 4 weeks, 18 g) used in this study were purchased from Hunan SJA Laboratory Animal Co. and were housed in the SPF standardized animal laboratory at the Animal Center of Hunan Normal University and fed a standard diet, under pathogen-specific conditions in a constant temperature chamber with 12 h of alternating light and dark. After allowed 1 week to acclimate to their surroundings, Mice were randomly grouped (*n* = 10 in each group). GINS2-KO or corresponding control cells were injected subcutaneously into each mouse (1 × 10^6^ cells/mouse). After 7 days, mice injected with the same cells and bearing tumor around 50 mm^3^ were subjected to TMZ or DMSO treatment (*n* = 5 in each group). The TMZ group received an intraperitoneal injection of 20 mg/kg TMZ every 3 days, and the DMSO group received an equivalent DMSO injection. A protocol of 5 days of drug injection/2 days of drug discontinuation was adopted (4 cycles in total). Tumor volumes were measured every 3 days with vernier calipers and calculated using the formula: volume (mm^3^) = length × width^2 ^× *π*/6. After treatment, all mice were euthanized and tumors were carefully removed, weighed, and photographed.

### RNA sequencing and data analysis

Total RNA of the target cells was collected and sent to Majorbio Biopharmaceutical Technology (Shanghai, China) for RNA expression profiling. DEGs between treated and control groups were screened using the “edgeR” R package (|FC| > 2, *P* < 0.05).

### Construction of TFs regulatory networks

The TFs regulatory network of upregulated genes (|FC| > 2) in SHG44-GINS2 was constructed by Network Analyst (https://www.networkanalyst.ca) based on ChIP Enrichment Analysis (ChEA) database. Subsequently, we screened the possible pathways starting from GINS2 and ending at ECT2, and explored the potential TFs linking GINS2 to ECT2.

### Co-immunoprecipitation (Co-IP) assay and LC-MS/MS analysis

Protein lysate was derived from U251 cells and bicinchoninic acid assay (BCA, E112-01, Vazyme) was performed for protein quantification. 10% protein was taken as Input. 10 μL washed Protein A + G beads (P2108, Beyotime) were added to the remaining protein lysate and incubated for 1 h at 4 °C with turning to remove non-specific impurities. For each 500 μL sample, 4 μg of anti-GINS2 antibody or normal IgG was added and incubated overnight at 4 °C. 20 μL of magnetic beads were used to adsorb antibody–protein complexes, and then the beads were washed three times with TBS. The beads were resuspended, and some were snap-frozen in liquid nitrogen for 5 min, then transferred to the refrigerator at −80 °C, and sent to Shanghai OE Biotech Co., Ltd (Shanghai, China) for LC-MS/MS protein identification. The remaining beads were denatured by adding 5 × Loading Buffer and water at 95 °C for 5 min, and the supernatant was used for Western blot.

### mRNA stability assay

The target cells were treated with 5 μg/mL of Actinomycin D (Act-D, M4881, AbMole) for 0, 0.5, 1, 2, 4 and 8 h. Total cell RNA was collected and extracted, and the mRNA expression of the target genes was detected by RT-qPCR assay.

### TF binding sites analysis

We used JASPAR (https://jaspar.genereg.net/) to predict the binding sites of EGR1 in the ECT2 promoter region and ChIP-Atlas (http://chip-atlas.org/) to visualize the relevant binding information in the ChIP-seq dataset.

### Predicting of protein–mRNA interaction

catRAPID (http://s.tartaglialab.com/page/catrapid_group) estimate the binding propensity of protein-RNA pairs by combining secondary structure, hydrogen bonding and van der Waals contributions [70]. Firstly, we used the catRAPID website to obtain 500 proteins that may bind to EGR1 mRNA, and intersected the 500 proteins with GINS2 interacting proteins. Subsequently, we calculated the interaction probabilities of the mRNA of the target gene EGR1 with these candidate proteins using the RNA Protein Interaction Prediction (RPISeq, http://pridb.gdcb.iastate.edu/RPISeq/references.php) website, which provides scores calculated by the Random Forest (RF) algorithm and the Support Vector Machine (SVM) algorithm. Predictions with a probability greater than 0.5 are considered positive, indicating that the corresponding RNAs and proteins may interact [71].

### ChIP assay

The specific steps of the ChIP are described previously [67]. Immunoprecipitation of cross-linked chromatin DNA was achieved with anti-EGR1 and normal IgG. qPCR detected the enrichment of EGR1 protein in the ECT2 promoter region. The primers used to detect EGR1 enrichment at the two predicted sites of the ECT2 promoter are shown in Supplementary Table 5.

### Dual-luciferase reporter assay

EGR1 overexpression/corresponding vector plasmid, reporter gene vector carrying the target site 1/2 sequence, and internal reference reporter gene vector were used to co-transfect 293 T cells. 24 h later, cells were washed three times, 100 μL ONE-GloTM detection reagent (E6110, Promega) was added to each well of 96-well plate, cells were incubated at room temperature for 10 min, and then the bioluminescence signal was detected. The experimental value = Firefly luciferase value/Renilla luciferase value*100%.

### Construction and validation of prognostic model

The R package required for the construction and validation of the prognostic model was mentioned in the previous study [66]. Briefly, we performed univariate Cox regression analysis and multifactor Cox regression analysis for three genes, GINS2, EGR1, and ECT2, based on sample information of TCGA-Glioma (*n* = 691). Risk score for each patient = GINS2 expression × GINS2 multifactor Cox regression coefficient + EGR1 expression × EGR1 multifactor Cox regression coefficient + ECT2 expression × ECT2 multifactor Cox regression coefficient. Next, patients were ranked according to risk scores, and median grouping generated low-risk and high-risk groups. A risk heat map, PCA, K-M survival analysis, Nomogram, and ROC curve were used to analyze the accuracy and specificity of the risk model. The CGGA sample (*n* = 590) was used as an external cohort for further validation of the prognostic model.

### Gene set variation analysis (GSVA)

We performed GSVA using the “GSVA” R package and the C2 gene set to measure each sample differential signaling pathway scores in high- and low-risk group based on gene expression, followed by a visual heat map using the “pheatmap” R package.

### Screening of GINS2 inhibitors

To screen for novel inhibitors of GINS2, TCGA glioma samples were divided into high and low GINS2 expression groups, and “edgeR” R package was performed to obtain DEGs. Subsequently, each of the 150 most significant up- and down-regulated genes was imported to the CMap database (https://clue.io/) to obtain the predicted GINS2 inhibitors and scores, with smaller scores indicating more significant mRNA inhibition of GINS2. The same method was used to analyze the glioma samples of CGGA. Finally, the results of TCGA and CGGA were combined using the R package “cmapR” to predict the potential inhibitors of GINS2.

### Statistical analysis

All experiments were repeated at least three times, and the results were expressed as mean ± SD. R software (version 4.2.0), GraphPad Prism 8 and *t*-test were used for statistical analysis, and *P* < 0.05 were considered statistically significant.

### Supplementary information

Supplementary Information

Uncropped western blots

Check list

Dataset 1

## Data Availability

The RNA-seq data used in this study is available in the Dataset 1. Expression profiles and clinical information of glioma clinical samples were downloaded from TCGA (https://portal.gdc.cancer.gov/) dataset and CGGA (http://www.cgga.org.cn/) dataset. All data that support the conclusions in this manuscript are available from the corresponding author upon reasonable request.

## References

[1] Ostrom QT, Price M, Neff C, Cioffi G, Waite KA, Kruchko C (2022). CBTRUS statistical report: primary brain and other central nervous system tumors diagnosed in the United States in 2015-2019. Neuro Oncol.

[2] Yang K, Wu Z, Zhang H, Zhang N, Wu W, Wang Z (2022). Glioma targeted therapy: insight into future of molecular approaches. Mol Cancer.

[3] Louis DN, Perry A, Wesseling P, Brat DJ, Cree IA, Figarella-Branger D (2021). The 2021 WHO classification of tumors of the central nervous system: a summary. Neuro Oncol.

[4] Friedman HS, Kerby T, Calvert H (2000). Temozolomide and treatment of malignant glioma. Clin Cancer Res.

[5] Gunther W, Pawlak E, Damasceno R, Arnold H, Terzis AJ (2003). Temozolomide induces apoptosis and senescence in glioma cells cultured as multicellular spheroids. Br J Cancer.

[6] Stupp R, Taillibert S, Kanner A, Read W, Steinberg D, Lhermitte B (2017). Effect of tumor-treating fields plus maintenance temozolomide vs maintenance temozolomide alone on survival in patients with glioblastoma: a randomized clinical trial. JAMA.

[7] Goldstein M, Kastan MB (2015). The DNA damage response: implications for tumor responses to radiation and chemotherapy. Annu Rev Med.

[8] Jackson SP, Bartek J (2009). The DNA-damage response in human biology and disease. Nature.

[9] Fu D, Calvo JA, Samson LD (2012). Balancing repair and tolerance of DNA damage caused by alkylating agents. Nat Rev Cancer.

[10] Lee SY (2016). Temozolomide resistance in glioblastoma multiforme. Genes Dis.

[11] Boskovic J, Coloma J, Aparicio T, Zhou M, Robinson CV, Mendez J (2007). Molecular architecture of the human GINS complex. EMBO Rep.

[12] Martinez MP, Wacker AL, Bruck I, Kaplan DL (2017). Eukaryotic replicative helicase subunit interaction with DNA and its role in DNA replication. Genes.

[13] Takayama Y, Kamimura Y, Okawa M, Muramatsu S, Sugino A, Araki H (2003). GINS, a novel multiprotein complex required for chromosomal DNA replication in budding yeast. Genes Dev.

[14] Chi F, Wang Z, Li Y, Chang N (2020). Knockdown of GINS2 inhibits proliferation and promotes apoptosis through the p53/GADD45A pathway in non-small-cell lung cancer. Biosci Rep.

[15] Liu X, Sun L, Zhang S, Zhang S, Li W (2020). GINS2 facilitates epithelial-to-mesenchymal transition in non-small-cell lung cancer through modulating PI3K/Akt and MEK/ERK signaling. J Cell Physiol.

[16] Zheng M, Zhou Y, Yang X, Tang J, Wei D, Zhang Y (2014). High GINS2 transcript level predicts poor prognosis and correlates with high histological grade and endocrine therapy resistance through mammary cancer stem cells in breast cancer patients. Breast Cancer Res Treat.

[17] Ouyang F, Liu J, Xia M, Lin C, Wu X, Ye L (2017). GINS2 is a novel prognostic biomarker and promotes tumor progression in early-stage cervical cancer. Oncol Rep.

[18] Zhang X, Zhong L, Liu BZ, Gao YJ, Gao YM, Hu XX (2013). Effect of GINS2 on proliferation and apoptosis in leukemic cell line. Int J Med Sci.

[19] Ye Y, Song YN, He SF, Zhuang JH, Wang GY, Xia W (2019). GINS2 promotes cell proliferation and inhibits cell apoptosis in thyroid cancer by regulating CITED2 and LOXL2. Cancer Gene Ther.

[20] Zhang Y, Hao X, Han G, Lu Y, Chen Z, Zhang L (2022). E2F1-mediated GINS2 transcriptional activation promotes tumor progression through PI3K/AKT/mTOR pathway in hepatocellular carcinoma. Am J Cancer Res.

[21] Shen YL, Li HZ, Hu YW, Zheng L, Wang Q (2019). Loss of GINS2 inhibits cell proliferation and tumorigenesis in human gliomas. CNS Neurosci Ther.

[22] Cook DR, Rossman KL, Der CJ (2014). Rho guanine nucleotide exchange factors: regulators of Rho GTPase activity in development and disease. Oncogene.

[23] Fields AP, Justilien V (2010). The guanine nucleotide exchange factor (GEF) Ect2 is an oncogene in human cancer. Adv Enzym Regul.

[24] Tatsumoto T, Xie X, Blumenthal R, Okamoto I, Miki T (1999). Human ECT2 is an exchange factor for Rho GTPases, phosphorylated in G2/M phases, and involved in cytokinesis. J Cell Biol.

[25] Reinhardt HC, Yaffe MB (2013). Phospho-Ser/Thr-binding domains: navigating the cell cycle and DNA damage response. Nat Rev Mol Cell Biol.

[26] Cao C, Han P, Liu L, Tang Y, Tian S, Zhang K (2021). Epithelial cell transforming factor ECT2 is an important regulator of DNA double-strand break repair and genome stability. J Biol Chem.

[27] Liu L, Dai X, Yin S, Liu P, Hill EG, Wei W (2022). DNA-PK promotes activation of the survival kinase AKT in response to DNA damage through an mTORC2-ECT2 pathway. Sci Signal.

[28] Lamb J (2007). The Connectivity Map: a new tool for biomedical research. Nat Rev Cancer.

[29] Lamb J, Crawford ED, Peck D, Modell JW, Blat IC, Wrobel MJ (2006). The Connectivity Map: using gene-expression signatures to connect small molecules, genes, and disease. Science.

[30] Subramanian A, Narayan R, Corsello SM, Peck DD, Natoli TE, Lu X (2017). A next-generation connectivity map: L1000 platform and the first 1,000,000 profiles. Cell.

[31] Scott AT, Weitz M, Breheny PJ, Ear PH, Darbro B, Brown BJ (2020). Gene expression signatures identify novel therapeutics for metastatic pancreatic neuroendocrine tumors. Clin Cancer Res.

[32] Yang C, Zhang H, Chen M, Wang S, Qian R, Zhang L (2022). A survey of optimal strategy for signature-based drug repositioning and an application to liver cancer. Elife.

[33] Li G, Zhang C, Liang W, Zhang Y, Shen Y, Tian X (2021). Berberine regulates the Notch1/PTEN/PI3K/AKT/mTOR pathway and acts synergistically with 17-AAG and SAHA in SW480 colon cancer cells. Pharm Biol.

[34] Vengoji R, Atri P, Macha MA, Seshacharyulu P, Perumal N, Mallya K (2021). Differential gene expression-based connectivity mapping identified novel drug candidate and improved temozolomide efficacy for Glioblastoma. J Exp Clin Cancer Res.

[35] McCracken DJ, Celano EC, Voloschin AD, Read WL, Olson JJ (2016). Phase I trial of dose-escalating metronomic temozolomide plus bevacizumab and bortezomib for patients with recurrent glioblastoma. J Neurooncol.

[36] Tang JH, Yang L, Chen JX, Li QR, Zhu LR, Xu QF (2019). Bortezomib inhibits growth and sensitizes glioma to temozolomide (TMZ) via down-regulating the FOXM1-Survivin axis. Cancer Commun.

[37] Vengoji R, Macha MA, Nimmakayala RK, Rachagani S, Siddiqui JA, Mallya K (2019). Afatinib and temozolomide combination inhibits tumorigenesis by targeting EGFRvIII-cMet signaling in glioblastoma cells. J Exp Clin Cancer Res.

[38] Shan DD, Zheng QX, Chen Z (2022). Go-Ichi-Ni-San 2: a potential biomarker and therapeutic target in human cancers. World J Gastrointest Oncol.

[39] Vaddavalli PL, Schumacher B (2022). The p53 network: cellular and systemic DNA damage responses in cancer and aging. Trends Genet.

[40] Brown JS, O’Carrigan B, Jackson SP, Yap TA (2017). Targeting DNA repair in cancer: beyond PARP inhibitors. Cancer Discov.

[41] Xiang S, Reed DR, Alexandrow MG (2023). The CMG helicase and cancer: a tumor “engine” and weakness with missing mutations. Oncogene.

[42] Xuan F, Huang M, Zhao E, Cui H (2018). MINA53 deficiency leads to glioblastoma cell apoptosis via inducing DNA replication stress and diminishing DNA damage response. Cell Death Dis.

[43] Liu Q, Turner KM, Alfred Yung WK, Chen K, Zhang W (2014). Role of AKT signaling in DNA repair and clinical response to cancer therapy. Neuro Oncol.

[44] Beier D, Schulz JB, Beier CP (2011). Chemoresistance of glioblastoma cancer stem cells-much more complex than expected. Mol Cancer.

[45] Saito S, Liu XF, Kamijo K, Raziuddin R, Tatsumoto T, Okamoto I (2004). Deregulation and mislocalization of the cytokinesis regulator ECT2 activate the Rho signaling pathways leading to malignant transformation. J Biol Chem.

[46] Liu X, Zhang J, Ju S, Liu L, Sun Y, Guo L (2023). ECT2 promotes malignant phenotypes through the activation of the AKT/mTOR pathway and cisplatin resistance in cervical cancer. Cancer Gene Ther.

[47] Zhang Q, Cao C, Gong W, Bao K, Wang Q, Wang Y (2020). A feedforward circuit shaped by ECT2 and USP7 contributes to breast carcinogenesis. Theranostics.

[48] Zhi T, Jiang K, Xu X, Yu T, Zhou F, Wang Y (2019). ECT2/PSMD14/PTTG1 axis promotes the proliferation of glioma through stabilizing E2F1. Neuro Oncol.

[49] Chen Y, Tian P, Liu Y (2017). P53 and protein phosphorylation regulate the oncogenic role of epithelial cell transforming 2 (ECT2). Med Sci Monit.

[50] He D, Xiang J, Li B, Liu H (2016). The dynamic behavior of Ect2 in response to DNA damage. Sci Rep.

[51] Qiu Y, Hu W, Wen M, Zhao W, Xie J, Zhang J (2022). Low expression of ECT2 confers radiation therapy resistance through transcription coupled nucleolar DNA damage repair. Int J Radiat Oncol Biol Phys.

[52] Van Nostrand EL, Freese P, Pratt GA, Wang X, Wei X, Xiao R (2020). A large-scale binding and functional map of human RNA-binding proteins. Nature.

[53] Wang B, Guo H, Yu H, Chen Y, Xu H, Zhao G (2021). The role of the transcription factor EGR1 in cancer. Front Oncol.

[54] Lv D, Gimple RC, Zhong C, Wu Q, Yang K, Prager BC (2022). PDGF signaling inhibits mitophagy in glioblastoma stem cells through N(6)-methyladenosine. Dev Cell.

[55] Chen L, Liu YC, Zheng YY, Xu J, Zhang Y, Liu WL (2019). Furanodienone overcomes temozolomide resistance in glioblastoma through the downregulation of CSPG4-Akt-ERK signalling by inhibiting EGR1-dependent transcription. Phytother Res.

[56] Madabhushi R, Gao F, Pfenning AR, Pan L, Yamakawa S, Seo J (2015). Activity-induced DNA breaks govern the expression of neuronal early-response genes. Cell.

[57] Shin SY, Kim CG, Lee YH (2013). Egr-1 regulates the transcription of the BRCA1 gene by etoposide. BMB Rep.

[58] Finn RS, Martin M, Rugo HS, Jones S, Im SA, Gelmon K (2016). Palbociclib and letrozole in advanced breast cancer. N Engl J Med.

[59] Whittaker S, Madani D, Joshi S, Chung SA, Johns T, Day B (2017). Combination of palbociclib and radiotherapy for glioblastoma. Cell Death Discov.

[60] Hashizume R, Zhang A, Mueller S, Prados MD, Lulla RR, Goldman S (2016). Inhibition of DNA damage repair by the CDK4/6 inhibitor palbociclib delays irradiated intracranial atypical teratoid rhabdoid tumor and glioblastoma xenograft regrowth. Neuro Oncol.

[61] Li Z, Zhang J, Zheng H, Li C, Xiong J, Wang W (2019). Modulating lncRNA SNHG15/CDK6/miR-627 circuit by palbociclib, overcomes temozolomide resistance and reduces M2-polarization of glioma-associated microglia in glioblastoma multiforme. J Exp Clin Cancer Res.

[62] Im JS, Ki SH, Farina A, Jung DS, Hurwitz J, Lee JK (2009). Assembly of the Cdc45-Mcm2-7-GINS complex in human cells requires the Ctf4/And-1, RecQL4, and Mcm10 proteins. Proc Natl Acad Sci USA.

[63] Zhang M, He S, Ma X, Ye Y, Wang G, Zhuang J (2020). GINS2 affects cell viability, cell apoptosis, and cell cycle progression of pancreatic cancer cells via MAPK/ERK pathway. J Cancer.

[64] Tatake RJ, O’Neill MM, Kennedy CA, Wayne AL, Jakes S, Wu D (2008). Identification of pharmacological inhibitors of the MEK5/ERK5 pathway. Biochem Biophys Res Commun.

[65] Park SJ, Choi YS, Lee S, Lee YJ, Hong S, Han S (2016). BIX02189 inhibits TGF-beta1-induced lung cancer cell metastasis by directly targeting TGF-beta type I receptor. Cancer Lett.

[66] He H, Liang L, Huang J, Jiang S, Liu Y, Sun X (2022). KIF20A is associated with clinical prognosis and synergistic effect of gemcitabine combined with ferroptosis inducer in lung adenocarcinoma. Front Pharm.

[67] Cong L, Xie X, Liu S, Xiang L, Fu X (2022). Genistein promotes M1 macrophage apoptosis and reduces inflammatory response by disrupting miR-21/TIPE2 pathway. Saudi Pharm J.

[68] Liang L, Yan B, Liu Y, Jiang S, He H, Huang J (2022). FOXP3 contributes to TMZ resistance, prognosis, and immune infiltration in GBM from a novel pyroptosis-associated risk signature. Dis Markers.

[69] Liang L, He H, Jiang S, Liu Y, Huang J, Sun X (2022). TIAM2 contributes to osimertinib resistance, cell motility, and tumor-associated macrophage M2-like polarization in lung adenocarcinoma. Int J Mol Sci.

[70] Armaos A, Colantoni A, Proietti G, Rupert J, Tartaglia GG (2021). catRAPID omics v2.0: going deeper and wider in the prediction of protein–RNA interactions. Nucleic Acids Res.

[71] Muppirala UK, Honavar VG, Dobbs D (2011). Predicting RNA-protein interactions using only sequence information. BMC Bioinforma.

