# Supplementary material for: GINS2 regulates temozolomide chemosensitivity via the EGR1/ECT2 axis in gliomas
Source: Cell Death Dis. 2024 Mar 11;15(3):205. doi: 10.1038/s41419-024-06586-w (PMC10928080; doi:10.1038/s41419-024-06586-w)
Supplement: Supplementary file 1 — Supplementary Information [file 41419_2024_6586_MOESM1_ESM.docx]

**Supplementary information for**

**GINS2 Regulates Temozolomide Chemosensitivity via the EGR1/ECT2 Axis in Gliomas**

Hua He, Lu Liang, *et al.*

*Corresponding author: Yiqun Jiang, jiangyiqun@hunnu.edu.cn (Lead Contact)

Li Cong, [congli@hunnu.edu.cn](mailto:congli@hunnu.edu.cn)

**
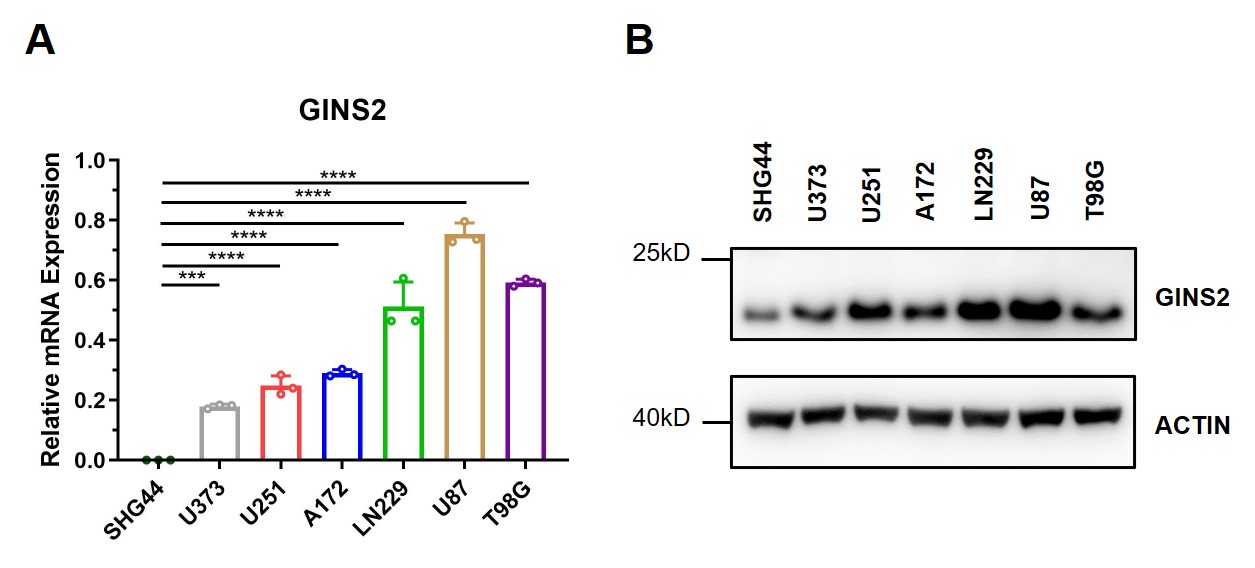
**

**Supplementary Figure 1. GINS2 is generally expressed at high levels in glioma cell lines. (A)** RT-qPCR to detect the mRNA expression level of GINS2 in glioma cell lines. **(B)** Western blot to detect the protein expression level of GINS2 in glioma cell lines. ****P* < 0.001, *****P* < 0.0001. n = 3 independent experiments. Two-Tailed *t*-Test Assuming Equal Variances. Error bars represent the mean +/− standard deviation of the mean.

**
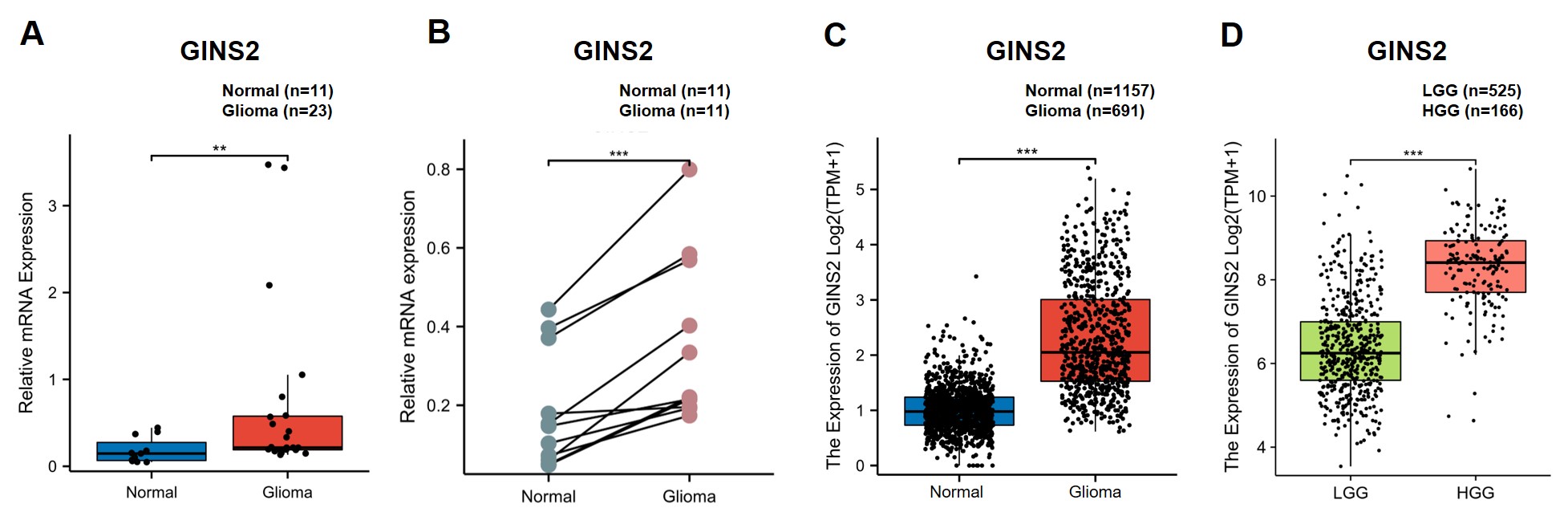
**

**Supplementary Figure 2. GINS2 is expressed higher in glioma than in normal tissues and correlates with tumor grade.** **(A)** RT-qPCR to detect the mRNA expression levels of GINS2 in 23 glioma tissues (right, red) and 11 paraneoplastic tissues (left, blue). **(B)** RT-qPCR to detect the mRNA expression levels of GINS2 in 11 paired glioma tissues (right, pink) and adjacent normal brain tissues (left, blue-green). **(C)** Bioinformatics analysis of GINS2 mRNA levels in glioma samples (right, red) and normal brain tissue samples (left, blue) from public databases. **(D)** Bioinformatics analysis of the difference in mRNA expression of GINS2 in LGG samples (left, green) and HGG samples (right, red) from TCGA database. LGG: Low-grade glioma, HGG: High-grade glioma. In the boxplot, center line as the median, the upper and lower boundaries represent the first and third quartiles, while whiskers extend to 1.5× the interquartile range. ***P* < 0.01, ****P* < 0.001. n = 3 independent experiments. Two-Tailed *t*-Test Assuming Equal Variances.

**
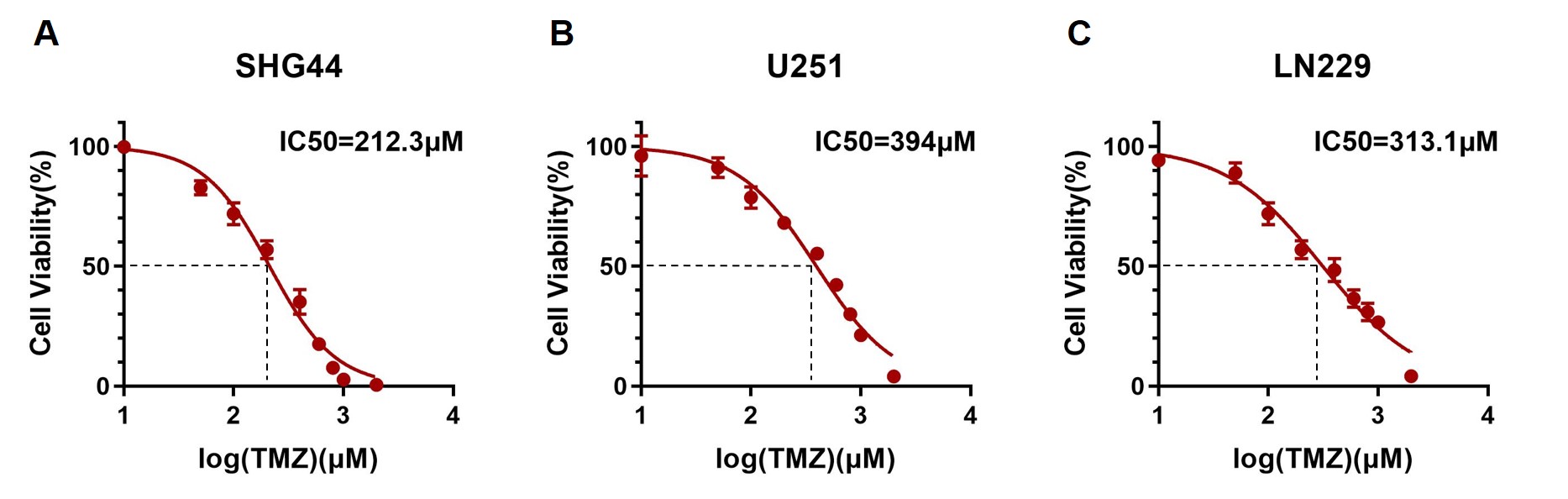
**

**Supplementary Figure 3. IC50 of TMZ in glioma cell lines SHG44, U251, LN229. (A)** CCK8 assay to detect the IC50 of TMZ in SHG44 cell line. **(B)** CCK8 assay to detect the IC50 of TMZ in U251 cell line. **(C)** CCK8 assay to detect the IC50 of TMZ in LN229 cell line. IC50: Half maximal inhibitory concentration. n = 3 independent experiments. Two-Tailed *t*-Test Assuming Equal Variances. Error bars represent the mean +/− standard deviation of the mean.

**
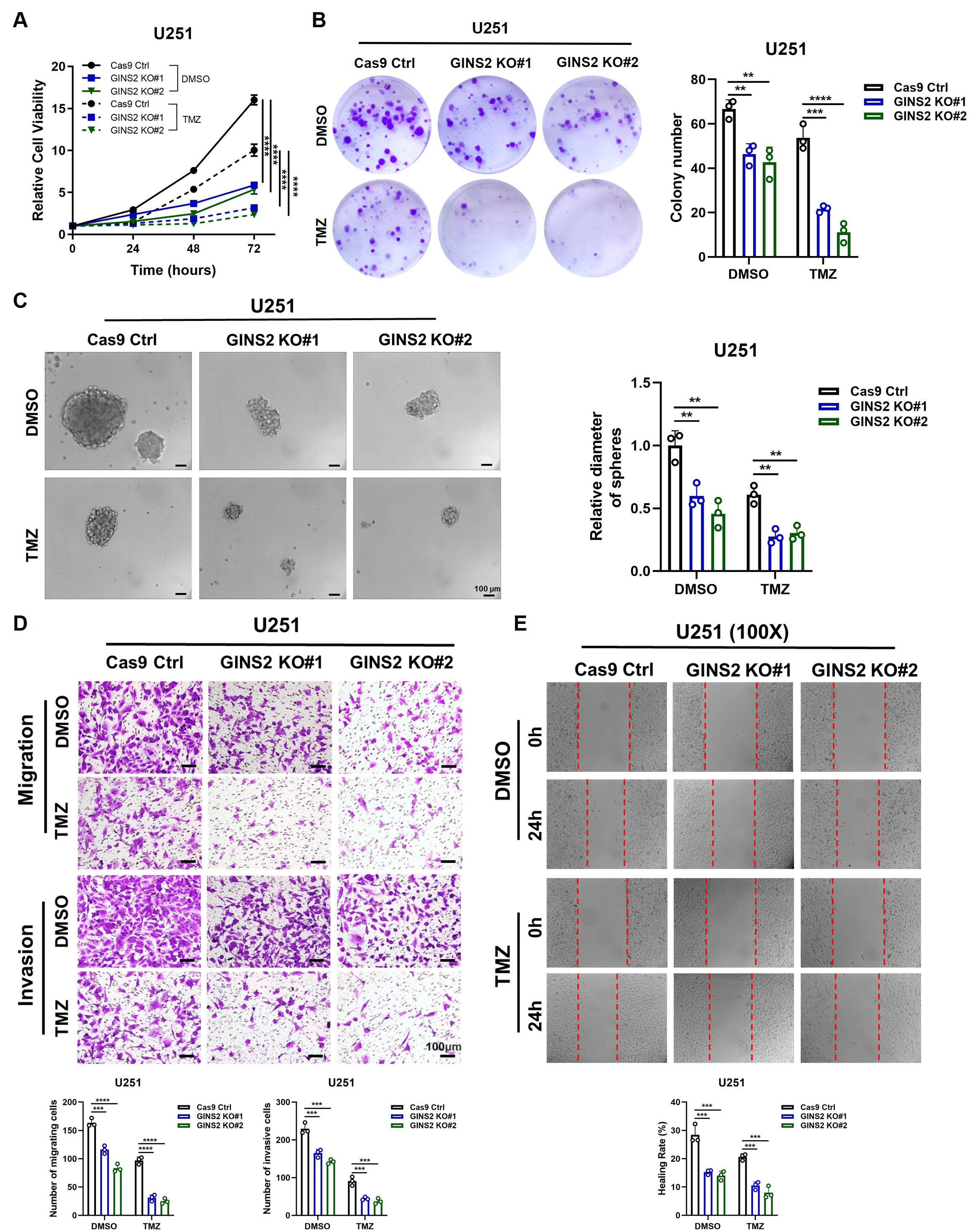
**

**Supplementary Figure 4. Knockout of GINS2 down-regulates the malignant phenotype and promotes the TMZ sensitivity of U251 cells. (A)** CCK8 assays were used to examine the effect of GINS2 on U251 cell proliferation and TMZ sensitivity. **(B)** GINS2 regulates clonogenic ability of U251 cells. **(C)** Stable knockout of GINS2 affects glioma cell stemness. **(D)** GINS2 promotes the migration and invasive ability of glioma cells. **(E)** Scratch assay to detect the effect of GINS2 on the migration ability of U251 cells. ***P* < 0.01, ****P* < 0.001, *****P* < 0.0001. n = 3 independent experiments.Two-Tailed *t*-Test Assuming Equal Variances. Error bars represent the mean +/− standard deviation of the mean.


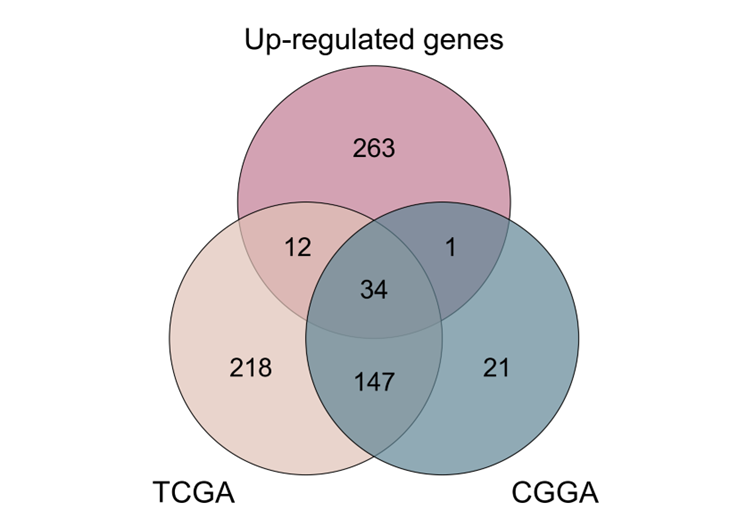


**Supplementary Figure 5.** Screening for genes strongly correlated with GINS2 expression based on RNA sequencing results, TCGA-Glioma and CGGA-Glioma.

**
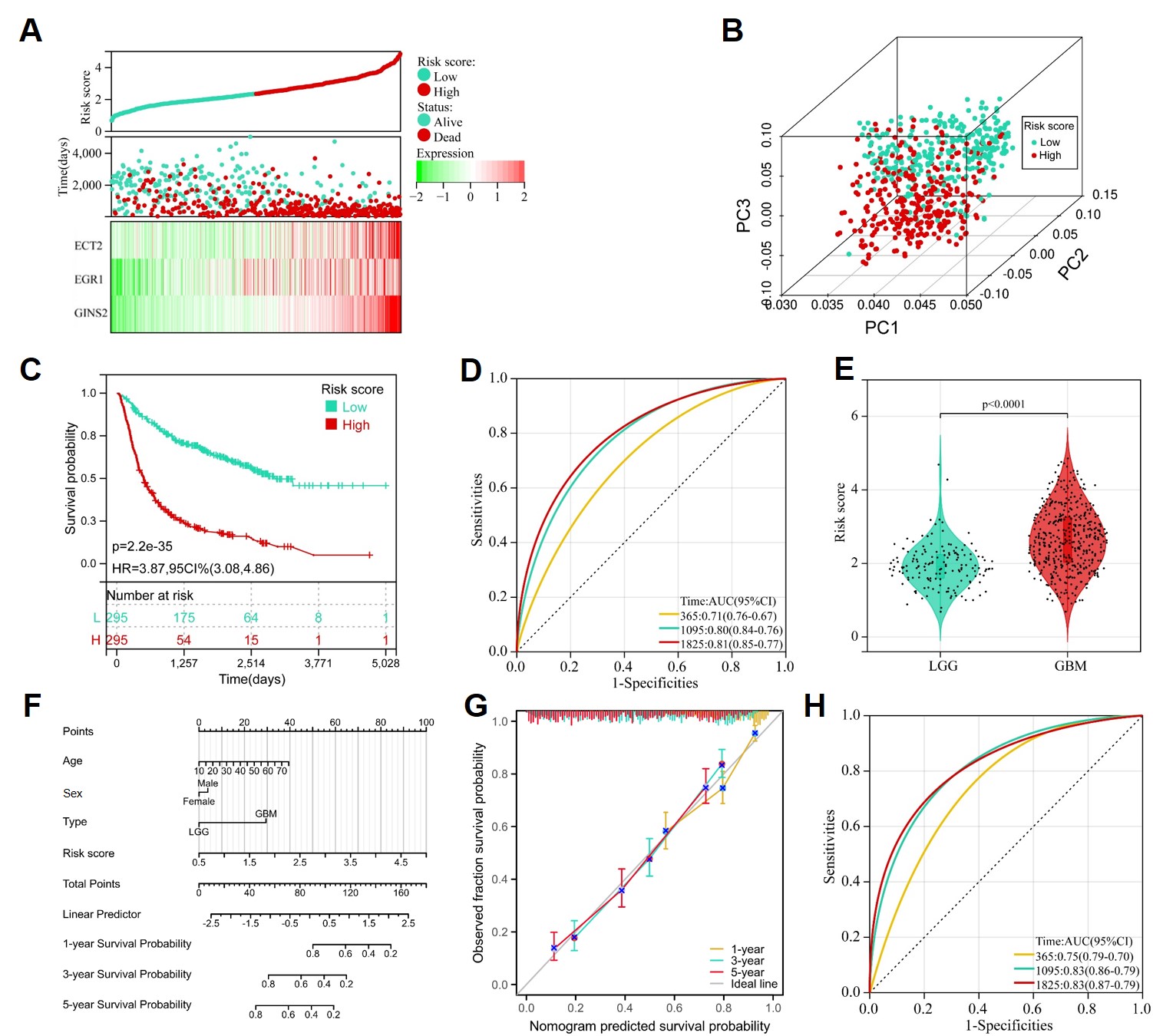
**

**Supplementary Figure 6. Validation of GEEPS based on CGGA glioma samples.** **(A)** Heat map of GINS2, EGR1, ECT2 gene expression, risk score curve and survival status scatter plot of glioma patients. **(B)** PCA analysis to determine the clustering performance of GEEPS. **(C)** K-M curves to analyze the difference in OS between glioma patients in the high-risk and low-risk groups. **(D)** ROC curves were used to verify the accuracy of GEEPS 1, 3 and 5-year survival prediction. **(E)** Comparison of risk scores between LGG patients (left, green) and HGG patients (right, red). In the violin plot, center line as the median, the upper and lower boundaries represent the first and third quartiles, while whiskers extend to 1.5× the interquartile range. **(F)** A Nomogram was constructed by combining four independent prognostic factors (risk score, age, sex, and tumor grade classification) to predict OS at 1, 3, and 5 years in glioma patients. **(G)** Calibration plots to validate the accuracy of the Nomogram. **(H)** ROC curves to verify the accuracy of survival prediction of the Nomogram. GEEPS: GINS2-EGR1-ECT2 pathway signature, PCA: Principal component analysis, K-M: Kaplan-Meier; ROC: Receiver operating characteristic curve, AUC: Area under the curve, LGG: Low-grade glioma, HGG: High-grade glioma. Two-Tailed *t*-Test Assuming Equal Variances. Error bars represent the mean +/− standard deviation of the mean.


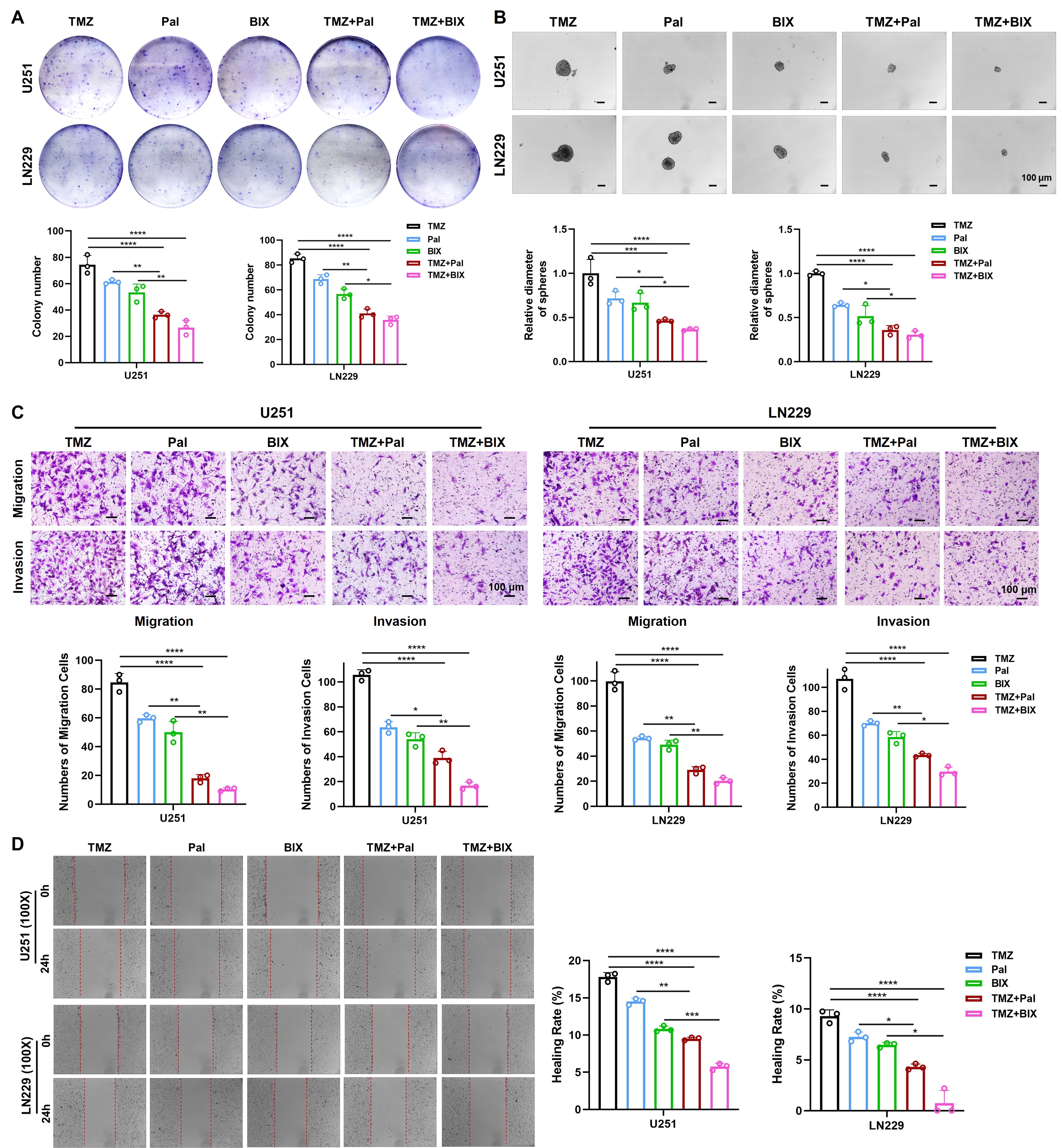


**Supplementary Figure 7. Pal/BIX and TMZ combination inhibit proliferation, stemness, invasion and migration capacity of glioma cells. (A)** Pal (20 μM)/BIX (20 μM) and TMZ (200 μM) decreases the clonogenic ability of U251 and LN229. **(B)** GINS2 inhibitor Pal (20 μM)/BIX (20 μM) and TMZ (200 μM) decreases the glioma cell stemness. **(C-D)** Pal (20 μM)/BIX (20 μM) and TMZ (200 μM) decreases the migration and invasive ability of glioma cells. Pal: Palbociclib; BIX: BIX-02189. **P* < 0.05, ***P* < 0.01, ****P* < 0.001, *****P* < 0.0001. n = 3 independent experiments. Two-Tailed *t*-Test assuming equal variances. Error bars represent the mean +/− standard deviation of the mean.

**Supplementary Table 1.** **sgRNA targets Sequences**

| **sgRNA** | **Oligo** |
| --- | --- |
| GINS2 KO#1 | TTCGTAAGTTCCATGTAGTA |
| GINS2 KO#2 | TCGTGCGGAGTTTGTACATG |
| GINS2 KO#3 | GATCTACTTACTGATTTAAC |

**Supplementary Table 2. RT-qPCR Primer Sequences**

| **Gene** | **Forward primer**  **（5'-3'）** | **Reverse primer**  **（5'-3'）** | **Product size（bp）** |
| --- | --- | --- | --- |
| GINS2 | AGCCAAACTCCGAGTGTCTGCT | CTTGTGTGAGGAAAGTCCCGCT | 113 |
| ECT2 | GCAGTCAGCAAGGTGGCAAGTT | CTCTGGTGCAAGGATAGGTCCA | 142 |
| EGR1 | AGCAGCACCTTCAACCCTCAGG | GAGTGGTTTGGCTGGGGTAACT | 133 |
| β-Actin | CACCATTGGCAATGAGCGGTTC | AGGTCTTTGCGGATGTCCACGT | 230 |

**Supplementary Table 3. 10 proteins interacting with GINS2 in mass spectrometry results**

| **proteins** | **Protein FDR Confidence: Combined** | **Peptides-GINS2** | **PSMs** | **Unique Peptides** | **Peptides-IgG** |
| --- | --- | --- | --- | --- | --- |
| DHX9 | High | 18 | 18 | 18 | 0 |
| RPL11 | High | 5 | 6 | 5 | 0 |
| RPS27A | High | 7 | 9 | 7 | 0 |
| SF3B3 | High | 15 | 15 | 15 | 0 |
| RPS17 | High | 7 | 9 | 7 | 0 |
| NPM1 | High | 9 | 15 | 9 | 0 |
| RPL28 | High | 6 | 6 | 6 | 0 |
| RPL13A | High | 6 | 7 | 6 | 0 |
| HNRNPA0 | High | 9 | 12 | 7 | 1 |
| RPS5 | High | 6 | 8 | 6 | 1 |

PSMs: Peptide-spectrum matches.

**Supplementary Table 4. Prediction of EGR1 mRNA and candidate protein binding probability**

| **Candidate proteins** | **RF** | **SVM** |
| --- | --- | --- |
| DHX9 | 0.7 | 0.59 |
| SF3B3 | 0.65 | 0.38 |
| NPM1 | 0.5 | 0.29 |
| RPL13A | 0.7 | 0.22 |
| RPL28 | 0.6 | 0.25 |
| RPS17 | 0.65 | 0.34 |
| RPS27A | 0.65 | 0.38 |
| RPL11 | 0.7 | 0.49 |
| RPS5 | 0.75 | 0.11 |
| HNRNPA0 | 0.7 | 0.14 |

RF: Random Forest; SVM: Support Vector Machines.

| **Site** | **Forward primer**  **（5'-3'）** | **Reverse primer**  **（5'-3'）** | **Product size（bp）** |
| --- | --- | --- | --- |
| Site 1 | TTATCTCTGGCGGCACTGATT | TGGTGGCTCCTGAGGTCTT | 180 |
| Site 2 | AAAGAGGTTGACATCACGCG | CCACTTGCACCTCTCTCACT | 190 |

**Supplementary Table 5. Primers for ChIP**

| **Pal Dose (μM)** | **TMZ Dose (μM)** | **Effect** | **CI** |
| --- | --- | --- | --- |
| 2.5 | 200.0 | 0.495 | 0.776 |
| 5.0 | 200.0 | 0.453 | 0.771 |
| 10.0 | 200.0 | 0.423 | 0.871 |
| 15.0 | 200.0 | 0.368 | 0.814 |
| 20.0 | 200.0 | 0.299 | 0.660 |
| 25.0 | 200.0 | 0.290 | 0.712 |
| 30.0 | 200.0 | 0.260 | 0.663 |
| 40.0 | 200.0 | 0.260 | 0.801 |
| 50.0 | 200.0 | 0.233 | 0.781 |
| 2.5 | 400.0 | 0.414 | 1.034 |
| 5.0 | 400.0 | 0.369 | 0.936 |
| 10.0 | 400.0 | 0.320 | 0.861 |
| 15.0 | 400.0 | 0.294 | 0.848 |
| 20.0 | 400.0 | 0.262 | 0.787 |
| 25.0 | 400.0 | 0.234 | 0.727 |
| 30.0 | 400.0 | 0.212 | 0.680 |
| 40.0 | 400.0 | 0.167 | 0.555 |
| 50.0 | 400.0 | 0.137 | 0.473 |
| 2.5 | 600.0 | 0.300 | 0.949 |
| 5.0 | 600.0 | 0.272 | 0.874 |
| 10.0 | 600.0 | 0.243 | 0.818 |
| 15.0 | 600.0 | 0.235 | 0.841 |
| 20.0 | 600.0 | 0.207 | 0.760 |
| 25.0 | 600.0 | 0.173 | 0.641 |
| 30.0 | 600.0 | 0.151 | 0.570 |
| 40.0 | 600.0 | 0.123 | 0.484 |
| 50.0 | 600.0 | 0.086 | 0.341 |

**Supplementary Table 6. Effect and CI of Pal and TMZ combined treatment in U251**

TMZ: Temozolomide, Pal: Palbociclib; CI: Combination index.

**Supplementary Table 7. Effect and CI of Pal and TMZ combined treatment in LN229**

| **Pal Dose (μM)** | **TMZ Dose (μM)** | **Effect** | **CI** |
| --- | --- | --- | --- |
| 2.5 | 200.0 | 0.383 | 0.617 |
| 5.0 | 200.0 | 0.351 | 0.667 |
| 10.0 | 200.0 | 0.301 | 0.692 |
| 15.0 | 200.0 | 0.252 | 0.623 |
| 20.0 | 200.0 | 0.234 | 0.648 |
| 25.0 | 200.0 | 0.181 | 0.471 |
| 30.0 | 200.0 | 0.113 | 0.243 |
| 40.0 | 200.0 | 0.105 | 0.257 |
| 50.0 | 200.0 | 0.063 | 0.135 |
| 2.5 | 400.0 | 0.322 | 0.800 |
| 5.0 | 400.0 | 0.293 | 0.785 |
| 10.0 | 400.0 | 0.228 | 0.644 |
| 15.0 | 400.0 | 0.194 | 0.578 |
| 20.0 | 400.0 | 0.176 | 0.558 |
| 25.0 | 400.0 | 0.153 | 0.497 |
| 30.0 | 400.0 | 0.126 | 0.404 |
| 40.0 | 400.0 | 0.082 | 0.251 |
| 50.0 | 400.0 | 0.051 | 0.147 |
| 2.5 | 600.0 | 0.247 | 0.796 |
| 5.0 | 600.0 | 0.219 | 0.729 |
| 10.0 | 600.0 | 0.180 | 0.629 |
| 15.0 | 600.0 | 0.153 | 0.555 |
| 20.0 | 600.0 | 0.130 | 0.482 |
| 25.0 | 600.0 | 0.121 | 0.466 |
| 30.0 | 600.0 | 0.096 | 0.365 |
| 40.0 | 600.0 | 0.068 | 0.258 |
| 50.0 | 600.0 | 0.050 | 0.190 |

TMZ: Temozolomide, Pal: Palbociclib; CI: Combination index.

**Supplementary Table 8. Effect and CI of BIX and TMZ combined treatment in U251**

| **BIX Dose (μM)** | **TMZ Dose (μM)** | **Effect** | **CI** |
| --- | --- | --- | --- |
| 2.5 | 200.0 | 0.462 | 0.877 |
| 5.0 | 200.0 | 0.381 | 0.837 |
| 10.0 | 200.0 | 0.226 | 0.552 |
| 15.0 | 200.0 | 0.225 | 0.717 |
| 20.0 | 200.0 | 0.138 | 0.450 |
| 25.0 | 200.0 | 0.106 | 0.377 |
| 30.0 | 200.0 | 0.079 | 0.299 |
| 40.0 | 200.0 | 0.071 | 0.328 |
| 50.0 | 200.0 | 0.070 | 0.387 |
| 2.5 | 400.0 | 0.322 | 0.814 |
| 5.0 | 400.0 | 0.247 | 0.669 |
| 10.0 | 400.0 | 0.201 | 0.656 |
| 15.0 | 400.0 | 0.219 | 0.896 |
| 20.0 | 400.0 | 0.169 | 0.745 |
| 25.0 | 400.0 | 0.094 | 0.409 |
| 30.0 | 400.0 | 0.078 | 0.365 |
| 40.0 | 400.0 | 0.062 | 0.334 |
| 50.0 | 400.0 | 0.046 | 0.273 |
| 2.5 | 600.0 | 0.243 | 0.793 |
| 5.0 | 600.0 | 0.185 | 0.636 |
| 10.0 | 600.0 | 0.092 | 0.341 |
| 15.0 | 600.0 | 0.109 | 0.470 |
| 20.0 | 600.0 | 0.087 | 0.408 |
| 25.0 | 600.0 | 0.064 | 0.319 |
| 30.0 | 600.0 | 0.057 | 0.305 |
| 40.0 | 600.0 | 0.055 | 0.340 |
| 50.0 | 600.0 | 0.045 | 0.308 |

TMZ: Temozolomide, BIX: BIX-02189; CI: Combination index.

**Supplementary Table 9. Effect and CI of BIX and TMZ combined treatment in LN229**

| **BIX Dose (μM)** | **TMZ Dose (μM)** | **Effect** | **CI** |
| --- | --- | --- | --- |
| 2.5 | 200.0 | 0.393 | 0.795 |
| 5.0 | 200.0 | 0.360 | 0.962 |
| 10.0 | 200.0 | 0.265 | 0.883 |
| 15.0 | 200.0 | 0.233 | 0.961 |
| 20.0 | 200.0 | 0.202 | 0.957 |
| 25.0 | 200.0 | 0.172 | 0.893 |
| 30.0 | 200.0 | 0.135 | 0.721 |
| 40.0 | 200.0 | 0.091 | 0.520 |
| 50.0 | 200.0 | 0.061 | 0.361 |
| 2.5 | 400.0 | 0.322 | 0.911 |
| 5.0 | 400.0 | 0.293 | 0.979 |
| 10.0 | 400.0 | 0.228 | 0.916 |
| 15.0 | 400.0 | 0.194 | 0.905 |
| 20.0 | 400.0 | 0.176 | 0.939 |
| 25.0 | 400.0 | 0.153 | 0.891 |
| 30.0 | 400.0 | 0.126 | 0.768 |
| 40.0 | 400.0 | 0.082 | 0.525 |
| 50.0 | 400.0 | 0.051 | 0.331 |
| 2.5 | 600.0 | 0.269 | 0.976 |
| 5.0 | 600.0 | 0.222 | 0.873 |
| 10.0 | 600.0 | 0.184 | 0.850 |
| 15.0 | 600.0 | 0.153 | 0.791 |
| 20.0 | 600.0 | 0.132 | 0.750 |
| 25.0 | 600.0 | 0.103 | 0.612 |
| 30.0 | 600.0 | 0.069 | 0.408 |
| 40.0 | 600.0 | 0.053 | 0.347 |
| 50.0 | 600.0 | 0.025 | 0.159 |

TMZ: Temozolomide, BIX: BIX-02189; CI: Combination index.
