# Supplementary material for: GINS2 regulates temozolomide chemosensitivity via the EGR1/ECT2 axis in gliomas
Source: Cell Death Dis. 2024 Mar 11;15(3):205. doi: 10.1038/s41419-024-06586-w (PMC10928080; doi:10.1038/s41419-024-06586-w)
Supplement: Supplementary file 2 — Uncropped western blots [file 41419_2024_6586_MOESM2_ESM.pdf]

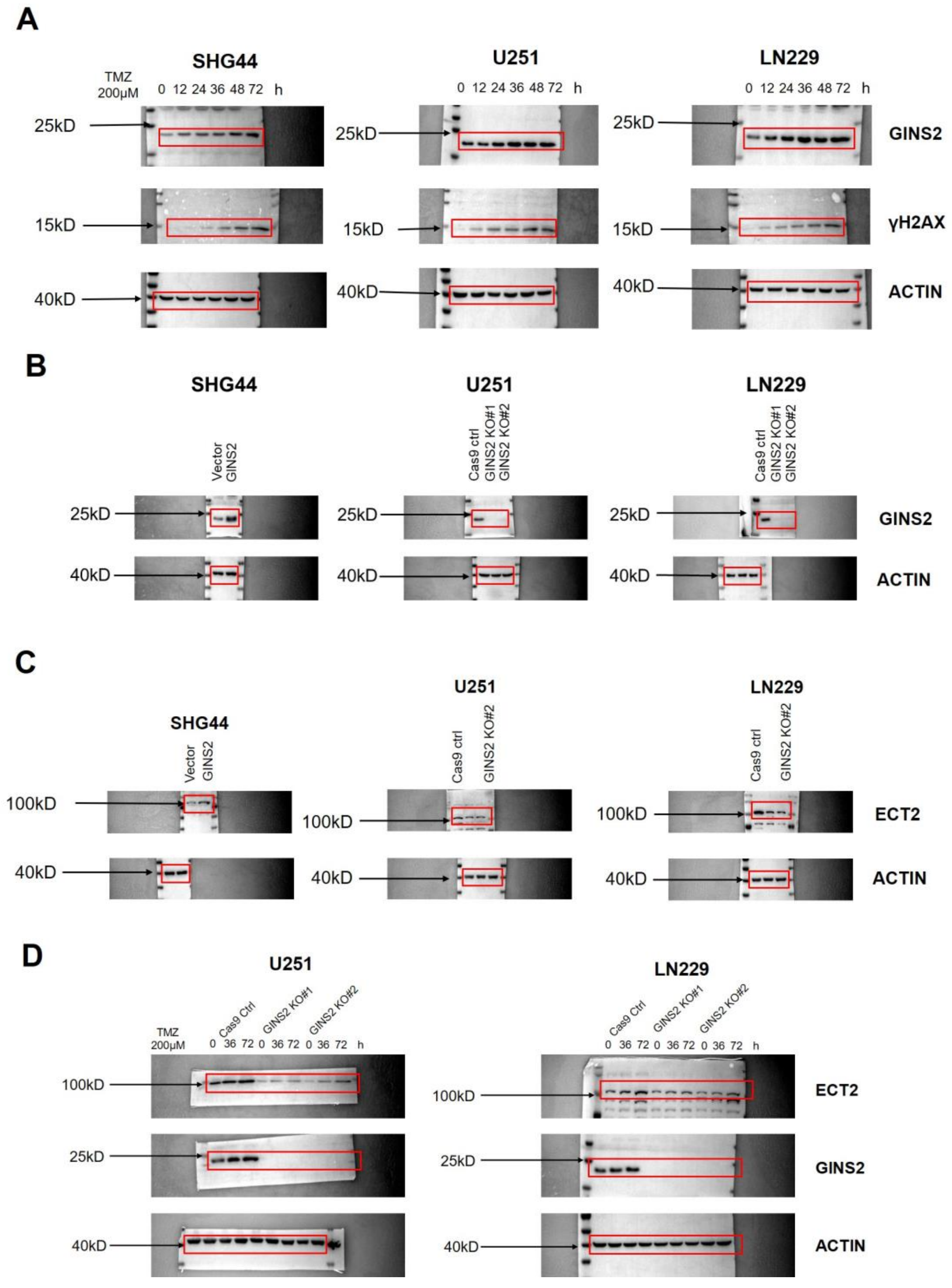
A
SHG44
U251
LN229
TMZ 200μM
0 12 24 36 48 72 h
25kD
15kD
40kD
GINS2
γH2AX
ACTIN
B
SHG44
U251
LN229
Vector
GINS2
Cas9 ctrl
GINS2 KO#1
GINS2 KO#2
25kD
40kD
GINS2
ACTIN
C
SHG44
U251
LN229
Vector
GINS2
Cas9 ctrl
GINS2 KO#2
100kD
40kD
ECT2
ACTIN
D
U251
LN229
TMZ 200μM
Cas9 Ctrl
GINS2 KO#1
GINS2 KO#2
0 36 72 0 36 72 0 36 72 h
100kD
25kD
40kD
ECT2
GINS2
ACTIN

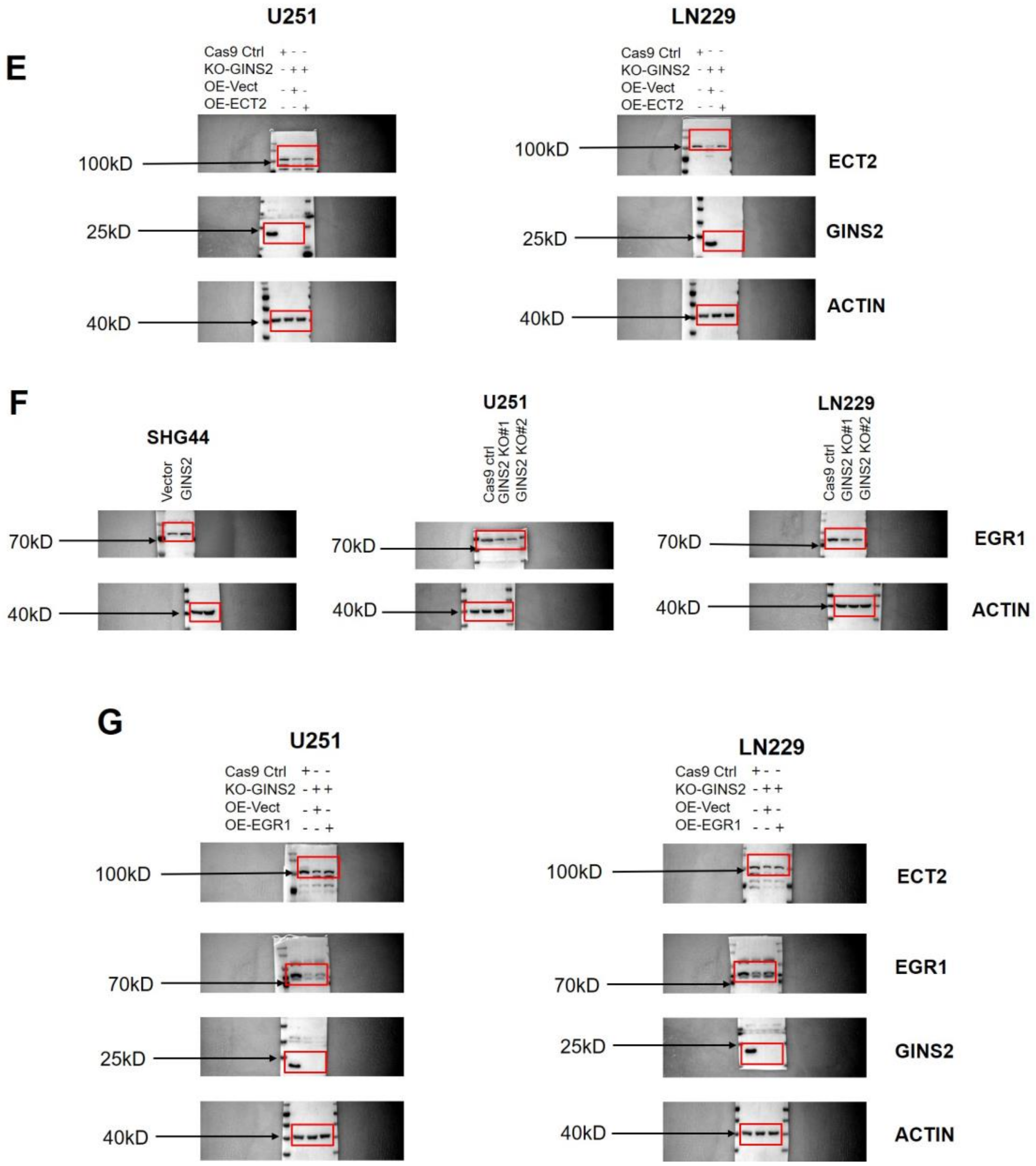
E
U251
Cas9 Ctrl + - -
KO-GINS2 - + +
OE-Vect - + -
OE-ECT2 - - +
100kD
25kD
40kD
LN229
Cas9 Ctrl + - -
KO-GINS2 - + +
OE-Vect - + -
OE-ECT2 - - +
100kD
25kD
40kD
ECT2
GINS2
ACTIN
F
SHG44
Vector
GINS2
70kD
40kD
U251
Cas9 ctrl
GINS2 KO#1
GINS2 KO#2
70kD
40kD
LN229
Cas9 ctrl
GINS2 KO#1
GINS2 KO#2
70kD
40kD
EGR1
ACTIN
G
U251
Cas9 Ctrl + - -
KO-GINS2 - + +
OE-Vect - + -
OE-EGR1 - - +
100kD
70kD
25kD
40kD
LN229
Cas9 Ctrl + - -
KO-GINS2 - + +
OE-Vect - + -
OE-EGR1 - - +
100kD
70kD
25kD
40kD
ECT2
EGR1
GINS2
ACTIN

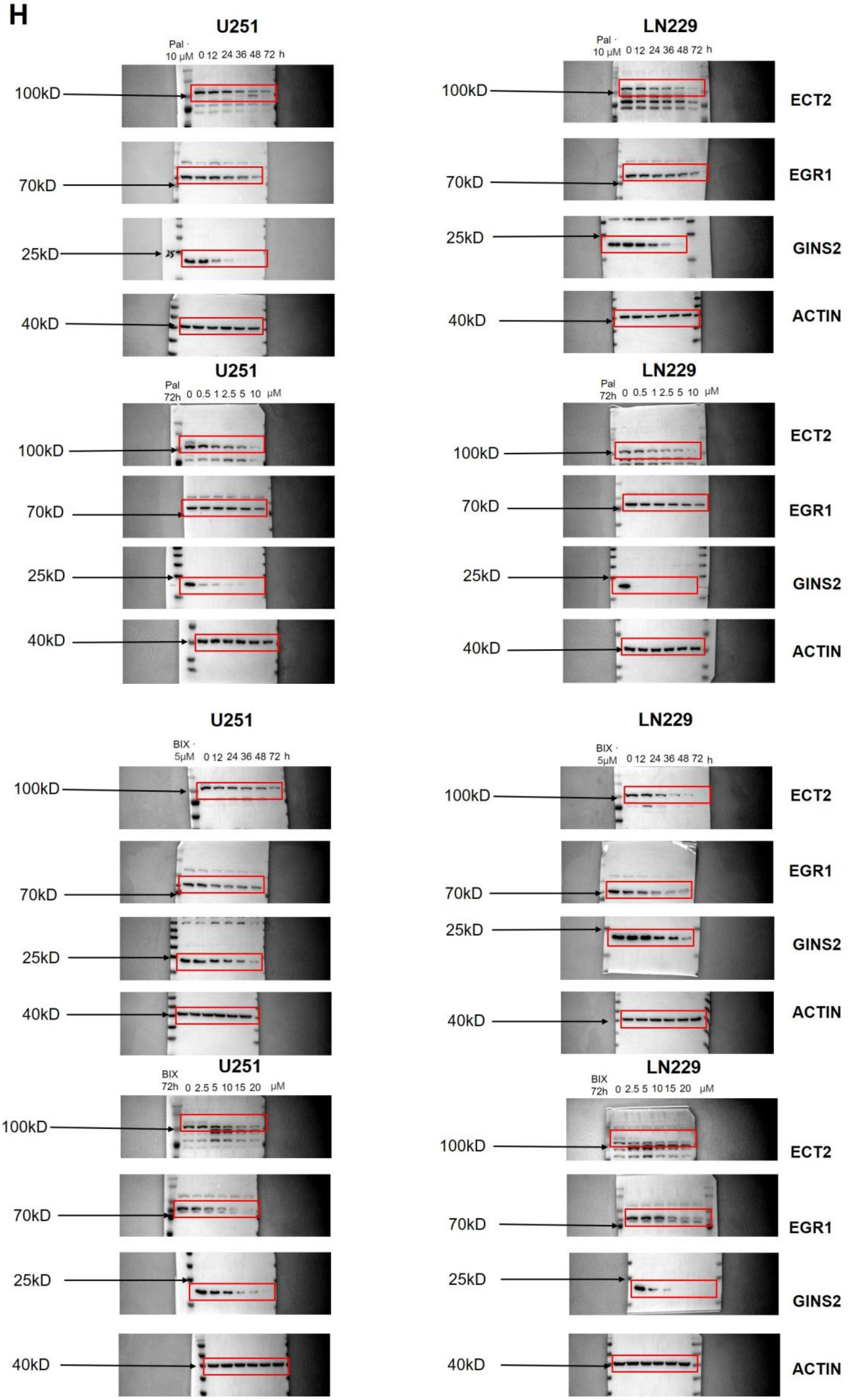
H
U251
LN229
Pal 10 μM 0 12 24 36 48 72 h
Pal 72h 0 0.5 1 2.5 5 10 μM
BIX 5μM 0 12 24 36 48 72 h
BIX 72h 0 2.5 5 10 15 20 μM
100kD
70kD
25kD
40kD
ECT2
EGR1
GINS2
ACTIN

I

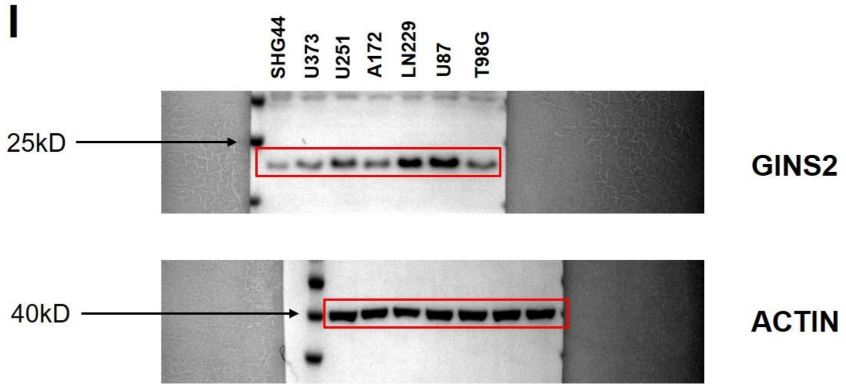

Uncropped western blots. **(A)** Uncropped western blots for main Figure 1A. **(B)** Uncropped western blots for main Figure 1C. **(C)** Uncropped western blots for main Figure 3E. **(D)** Uncropped western blots for main Figure 3F. **(E)** Uncropped western blots for main Figure 4A. **(F)** Uncropped western blots for main Figure 5B. **(G)** Uncropped western blots for main Figure 5E. **(H)** Uncropped western blots for main Figure 7C, D, E, F. **(I)** Uncropped western blots for main figure Supplementary Figure 1B.
